# Influenza coinfection inhibits control of mycobacterial infection in a human challenge model

**DOI:** 10.1038/s41467-026-72363-2

**Published:** 2026-06-11

**Authors:** Claire M. Broderick, Oliver Powell, Sam Nichols, Giselle D’Souza, Dominic Habgood-Coote, Carlota Miranda-Sole, Emily M. Whettlock, Zoe Gardener, Emma Bergstrom, Victoria J. Wright, Christopher W. Woods, Christopher Chiu, Sandra M. Newton, Elizabeth Whittaker, Michael Levin, Myrsini Kaforou

**Affiliations:** 1 Department of Infectious Disease, Imperial College London, London, UK; 2 Department of Medicine, Duke University Medical Center, Durham, NC USA; 3 Department of Paediatric Infectious Diseases, Imperial College Healthcare NHS Trust, London, UK

**Keywords:** Experimental models of disease, Tuberculosis, Influenza virus, Tuberculosis, Computational biology and bioinformatics

## Abstract

*Mycobacterium tuberculosis* infection is a dynamic continuum. Clinical outcomes reflect complex host-pathogen interactions. Epidemiological and animal studies have suggested influenza coinfection as a risk factor for progression from contained infection to active disease, but human studies have been lacking. Using a whole blood luminescent mycobacterial growth inhibition assay within a human influenza challenge study, we show that influenza infection reduces immunological control of mycobacterial growth. Transcriptome-wide RNA sequencing, cytokine and cellular analyses of subjects’ blood before and after influenza infection reveal that innate immune pathways, including type 1 interferon signalling, are activated by influenza but their subsequent responsiveness to mycobacteria is reduced, with multiple genes’ responses to BCG *lux* infection repressed by influenza coinfection. Our data suggest that influenza infection impairs immune mechanisms that contain mycobacterial growth and may be a risk factor for tuberculosis (TB) disease. Influenza vaccination might offer high risk, high prevalence populations protection against TB disease.

## Abstract (web summary)

Epidemiology suggests influenza coinfection may be a risk factor for tuberculosis disease. Using a human challenge study, the authors show that systemic influenza infection impairs immunological control of mycobacterial infection in healthy adults.

## Introduction

Tuberculosis (TB) remains a major cause of global morbidity and mortality^1^. Millions of people are exposed to and infected with *Mycobacterium tuberculosis* (*Mtb*) each year, yet the majority do not develop disease. Understanding the factors that drive progression to TB disease is essential for improving TB prevention and control strategies. *Mtb* infection is now recognised as a dynamic continuum, from quiescent latent infection, through to destructive, potentially life-threatening disease^2^. Outcomes after exposure are thought to reflect the continuing balance between the host’s immunological defences and mycobacterial virulence mechanisms. In addition to the well-established effects of malnutrition, HIV infection and other immunosuppressive conditions^2^, coinfection with other pathogens has attracted increasing interest as a contributing factor to progression to active disease^3^.

Humans are exposed to a range of pathogens throughout life, and there is growing evidence that the resulting immune responses may alter susceptibility to other infections. For example, influenza virus infection predisposes to secondary bacterial infections, including *Streptococcus pneumoniae* and *Staphylococcus aureus*, through a variety of mechanisms, including immunomodulation^4^. It has been postulated that concurrent influenza infection may also increase TB susceptibility and alter clinical outcomes after *Mtb* infection, through modulation of immunological pathways important in mycobacterial control^3^, with supporting evidence from epidemiological^5–9^ and animal model studies^10–13^. However, human studies have been limited, mostly observational and with mixed findings^14–16^. Given influenza is a widely circulating global pathogen (an estimated billion cases annually^17^), understanding its immunological interaction with *Mtb* may have implications for TB control, immunisation strategies and novel host-based TB diagnostics.

Mycobacterial growth inhibition assays (MGIAs) that measure the ability of whole blood or cells to inhibit mycobacterial growth in vitro allow a range of immunological components (cells, antibodies and cytokines) and their interactions to be considered in one model over time^18,19^. Human challenge studies provide a unique opportunity to observe pathogen-driven immune responses in a highly controlled environment. We employed a whole blood MGIA within the framework of a human influenza challenge study to investigate the effects of systemic influenza infection on host mycobacterial control. We utilised a recombinant reporter strain of mycobacteria, *Mycobacterium bovis* Bacille Calmette Guerin (BCG) *lux*, whose luminescence provides a quantitative read-out of live mycobacteria present^20,21^. Prior studies have demonstrated the BCG *lux* MGIA reflects differences in anti-mycobacterial immune responses from TB infection^22^, HIV infection^23^, BCG vaccination^24^, vitamin D^25^, neutrophil depletion^26^ and infliximab therapy^27^. Longitudinal RNA expression data from the model have provided insights into early immune responses to *Mtb*^28,29^.

In this work, we test the hypothesis that influenza infection of healthy adults alters whole blood mycobacterial control through modulation of anti-mycobacterial immune responses. Volunteers were challenged with the Influenza A virus and subsequent infection virologically confirmed. Blood aliquots, taken from participants before and after influenza infection, were infected with BCG *lux* and incubated. Whole blood mycobacterial growth and transcriptomic, cytokine and cellular responses to mycobacterial infection were measured in parallel and compared in the same subjects, before versus after influenza infection. We show that systemic influenza infection reduces whole blood control of mycobacteria via its disruption of innate immunological pathways, including type 1 interferon signalling. Our data suggest that influenza infection may be a risk factor for developing TB disease, and interventions such as the seasonal influenza vaccine that protect against influenza could offer protection against TB disease.

## Results

**Fig. 1:**
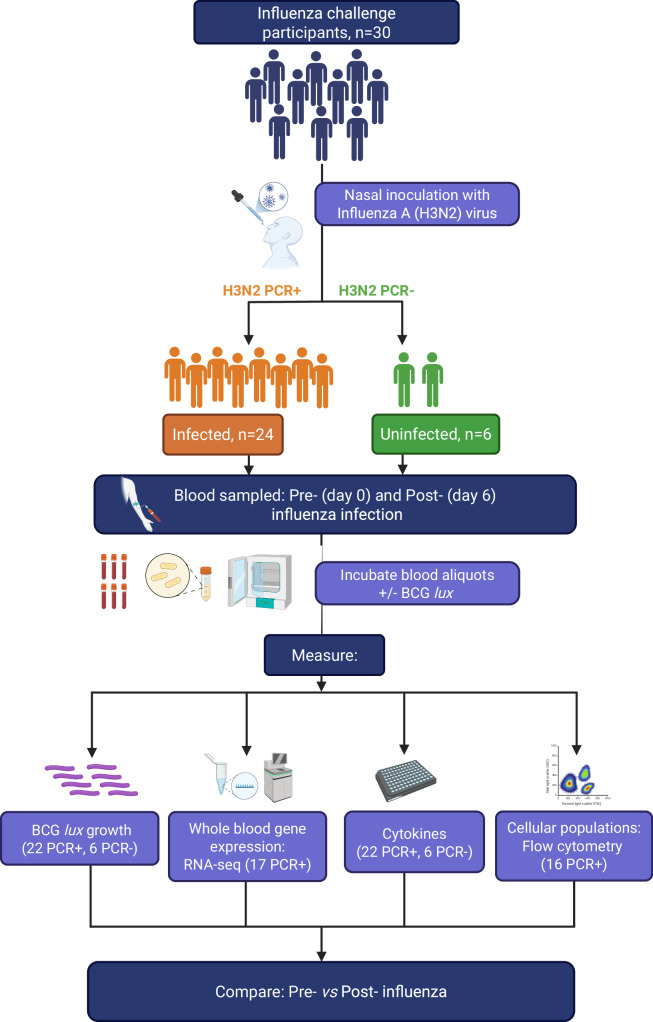
Study design and sampling. Thirty healthy adult participants were per nasally inoculated with Influenza A (H3N2) virus, with infection confirmed by qPCR of nasal lavage samples. Whole blood samples collected an hour prior to influenza inoculation (Day [D]0, pre-influenza) and 6 days after inoculation (D6, post-influenza) were incubated for 0 to 72 h, with and without BCG *lux*, for parallel measures of mycobacterial growth, transcriptomic, cytokine, and cellular responses. Influenza PCR + and PCR- participants were included in the BCG *lux* growth inhibition and cytokine studies, with 2 PCR + participants excluded from the analyses due to incomplete BCG *lux* growth data. For the RNA sequencing and flow cytometry studies, only PCR + participants with complete sets of blood aliquots available from the 0, 6 and 24 h timepoints were included. Created in BioRender. Broderick, C. (2025) https://BioRender.com/7jfia0k.

Thirty healthy adult participants were per nasally inoculated with Influenza A (subtype H3N2) virus, with 24 developing PCR-confirmed influenza infection (PCR-positive [+] group) and six remaining uninfected (PCR-negative [-]) group (Fig. 1). There were no significant differences in age, sex or ethnicity between the PCR + and PCR- groups (Supplementary Table 1). All participants had blood samples collected an hour prior to influenza inoculation (Day [D]0, pre-influenza) and 6 days after inoculation (D6, post-influenza), which were then incubated for 0 to 72 h, with and without BCG *lux* (Fig. 1).

### Influenza infection inhibits mycobacterial growth restriction

**Fig. 2:**
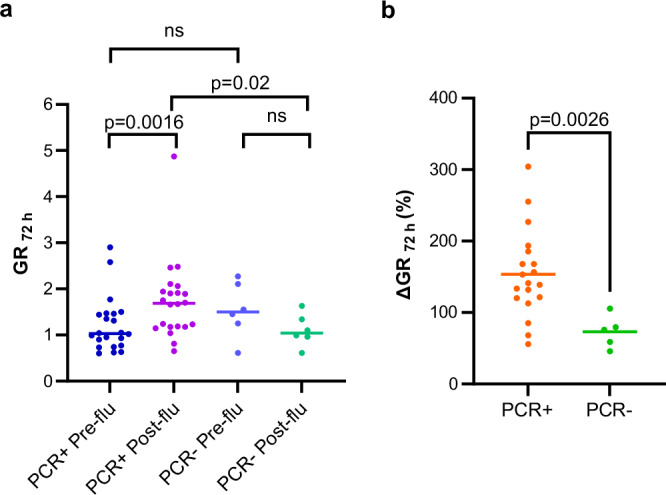
BCG *lux* Growth Ratios at 72 h. **a** Comparisons of whole blood BCG *lux* Growth Ratios after 72 h incubation (GR _72 h_) in 22 participants testing H3N2 influenza PCR + and 6 participants testing H3N2 influenza PCR-. **b** Change in GR_72 h_ (ΔGR_72 h_) in PCR + and PCR- participants. ΔGR_72 h_ was calculated as: (Post-influenza GR_72 h_ / Pre-influenza GR_72 h_) x 100% and compared between the PCR + versus PCR- participants. Comparisons of medians were made using two-tailed Wilcoxon rank sum, with paired comparisons for pre- versus post-influenza within both the PCR + and PCR- groups. ns denotes *p* > 0.05. Source data are provided as a Source Data file.

Firstly, we investigated the effect of systemic influenza infection on mycobacterial growth restriction by whole blood. Mycobacterial growth was measured as a Growth Ratio (GR_72 h_), with GR_72 h_ > 1 and < 1 indicating more and fewer BCG *lux* colonies at 72 h compared to baseline, respectively. The BCG *lux* GR_72 h_ in the influenza-infected (PCR + ) group was significantly higher in the blood sampled post-influenza inoculation (GR_72 h_ = 1.69) compared to pre-influenza inoculation (GR_72 h_ = 1.03 [paired analysis]), and also significantly higher than in post-influenza inoculation blood from the influenza-uninfected (PCR-) group (GR_72 h_ = 1.05), indicating reduced growth restriction following influenza infection. In the PCR- group, no significant differences in GR_72 h_ were observed pre- (GR_72 h_ = 1.5) versus post-influenza inoculation (GR_72 h_ = 1.05 [paired analysis]) (Fig. 2a). The change in GR_72 h_ following influenza inoculation compared to baseline (ΔGR_72 h_) was significantly higher in the PCR+ group versus the PCR- group (PCR + 147% *vs* PCR- 77%, Fig. 2b).

The GR_72 h_ and ΔGR_72 h_ did not differ significantly with sex or ethnicity or correlate with age. ΔGR_72 h_ also did not correlate significantly with maximal influenza viral load. (Supplementary Table 2).

### Host mycobacterial response genes switched on by influenza infection show reduced responses to BCG *lux*

Next, we sought to investigate the immunological factors that contributed to the increased growth of mycobacteria in whole blood following influenza infection. We took a hypothesis-free approach, starting with transcriptional analysis of whole blood RNA sequencing data from the MGIA. Paired data from 150 samples from 15 influenza PCR + participants were analysed and included BCG *lux*- infected and -uninfected whole blood from timepoints 0 h, 6 h and 24 h, collected pre- and post-influenza infection (Supplementary Fig. 1). Paired differential expression analyses were undertaken in DESeq2^30^, aiming to identify genes for which the transcriptomic response to BCG *lux* infection changed significantly in the presence versus absence of influenza infection. This was done through the inclusion of an interaction term between BCG and Influenza (BCG: Influenza) in the model.

**Fig. 3:**
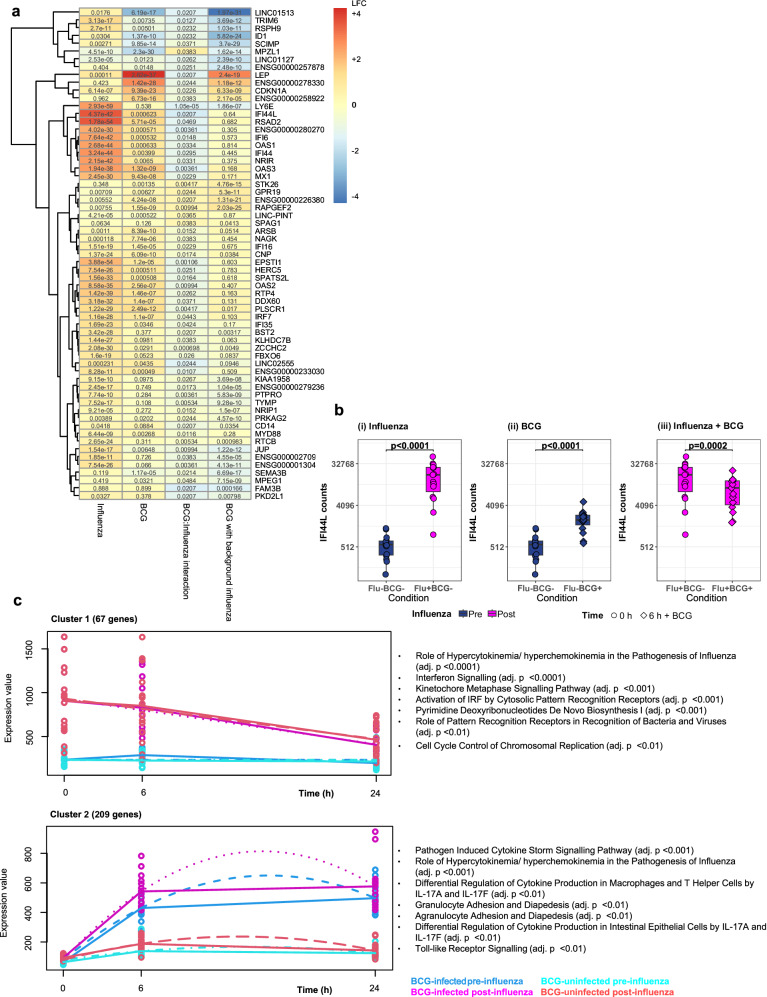
Transcriptomic responses to BCG *lux* altered by influenza. **a** Significantly differentially expressed genes (SDE) for the BCG: Influenza interaction. Differential expression analysis was performed using the DESeq2 package. Statistical significance was determined using a two-sided Wald test to assess whether the log2-fold change for a given comparison was different from zero. The resulting *p*-values were adjusted for multiple comparisons using the Benjamini-Hochberg (BH) method. Heat map representation of the 63 genes SDE for the BCG: Influenza interaction. For each of these genes, their log2 fold change (LFC) in response to Influenza, BCG, the BCG: Influenza interaction, and in response to BCG in the presence of background Influenza are shown. The associated BH-adjusted *p*- values (adj. p) are given in each cell. Where there is no gene name, the Ensembl ID is provided. **b** Effects of Influenza, BCG *lux* and their interaction on *IFI44L* gene expression. *IFI44L* was the SDE gene with the largest absolute LFC for the BCG: Influenza interaction term. Plots showing *IFI44L* expression (i) at baseline (0 h), before (dark blue circles) versus after (pink circles) influenza infection; (ii) in pre-influenza blood, at baseline (dark blue circles) versus after 6 h incubation with BCG *lux* (dark blue diamonds), (iii) in post-influenza blood, at baseline (pink circles) versus after 6 h incubation with BCG *lux* (pink diamonds). Box and whiskers plots are shown for 15 influenza PCR + participants, with the boxes representing the interquartile ranges (IQR), the horizontal lines showing the median expression values and whiskers denoting adjacent values within 1.5 IQR of the first and third quartiles. Paired two-tailed Wilcoxon rank sum was used for comparisons of median expression, with false discovery rate (FDR)-corrected *p*-values shown. **c** Gene clusters 1 and 2 identified from the maSigPro analysis. Analysis of time-course gene expression data was performed using the maSigPro package, which uses a two-step polynomial regression approach. Significance of temporal changes and differences between groups was assessed using an F-test. *P*-values from this test were adjusted for multiple comparisons using the BH method. In total, 2140 genes were identified as SDE over time and between groups (BH adj. *p* ≤ 0.05, R^2^ > 0.6). The coefficients obtained were used to group together SDE genes into clusters with similar temporal expression patterns. Summary plots of gene expression over time for gene clusters 1 and 2 with BCG-infected pre-influenza (dark blue), BCG-infected post-influenza (pink), BCG-uninfected pre-influenza (light blue) and BCG-uninfected post-influenza (red) groups shown. Solid lines connect the median expression to show the trends for each group, and the dashed lines show the regression curves fitted to the data. IPA was used for pathway analysis for each gene cluster. The top seven most significant pathways for clusters 1 and 2 are listed with their BH-adjusted *p*-values.

We identified 63 genes in which the transcriptomic response to BCG was significantly altered by influenza infection (Benjamini-Hochberg [BH]^31^ adjusted *p*-values [adj. *p*] ≤ 0.05 for BCG: Influenza) (Fig. 3a). Of these, 7 were increased after influenza infection (positive log2 fold change [LFC] for BCG: Influenza) and 56 had decreased expression (negative LFC for BCG: Influenza), meaning the expression of these genes in response to *BCG* was diminished in the presence of influenza infection (Fig. 3b). Many of these genes (e.g., *IFI6, IFI16, IFI35, IFI44, IFI44L, OAS1, OAS2, OAS3, RSAD2, DDX60, MX1*, *BST2*) were interferon (IFN) stimulated genes (ISGs), and most have immunological roles described (Supplementary Data 1). The significantly differentially expressed (SDE) genes for the variables BCG (12,358) and Influenza (*n* = 4761) are available in the supplementary (Supplementary Data 2, 3).

Having identified genes with significantly altered anti-mycobacterial responses in the presence of influenza infection, next we aimed to understand the biological processes affected by these genes by mapping them to biological pathways using Ingenuity Pathway Analysis (IPA; QIAGEN Inc., https://www.qiagenbioinformatics.com/products/ingenuity-pathway-analysis). The most significantly enriched canonical pathways (BH adj. *p* ≤ 0.05) for the 63 BCG: Influenza interaction genes were Role of Hypercytokinaemia/ hyperchemokinaemia in the Pathogenesis of Influenza (adj. *p* 2.14E-07, z-score − 2.6), Role of Pattern Recognition Receptors in Recognition of Bacteria and Viruses (adj. *p* 0.00013, z-score indeterminate) and Interferon Signalling (adj. *p* 0.00013, z-score = − 2). For two of these pathways, the z-score was negative, indicating the pathway was downregulated by the BCG: Influenza interaction (Supplementary Data 4).

The SDE genes and pathways identified for the variables Influenza and BCG (Supplementary Data 2, 3 and 5, 6) overlapped with those reported in previous studies of influenza^32,33^ and TB^28,29,34–36^.

To assess if the observed transcriptomic differences in gene expression were driven by differences in cell proportions, a second DESeq2 model including in silico estimated cell fractions was employed that gave similar results, suggesting that the transcriptomic differences observed for the BCG: Influenza interaction were driven by transcriptional regulation, rather than just differences in cell proportions. The cell-adjusted model identified 39 SDE genes for the BCG: Influenza interaction term, including 24 genes identified with the original model, with others just missing the significance threshold (e.g., *IFI44L* adj. *p* = 0.09, *IFI6* adj. *p* = 0.11, *IFI44* adj. *p* = 0.12, *OAS1* adj. *p* = 0.13) (Supplementary Data 7, and for the variables BCG and Influenza, Supplementary Data 8 and 9). As with the original model, these genes were enriched for Role of Pattern Recognition Receptors in Recognition of Bacteria and Viruses (adj. *p* 0.0060, z-score indeterminate) and Role of Hypercytokinaemia/ hyperchemokinaemia in the Pathogenesis of Influenza (adj. *p* 0.00069, z-score indeterminate) (Supplementary Data 10–12 for cell-adjusted IPA results).

To complement the pairwise DESeq2 analysis, a temporal model of the RNA sequencing data, to identify clusters of SDE genes with similar temporal expression patterns, was employed using maSigPro^37,38^. Designed specifically for time-course data, maSigPro may be more sensitive than conventional pairwise comparisons for analysis of whole blood longitudinal transcriptomic studies^39,40^. Four groups were defined in the model (Pre-influenza/BCG-uninfected; Pre-influenza/BCG-infected; Post-influenza/BCG-uninfected; Post-influenza/BCG-infected*)*. For the comparison of interest for this study (Pre-influenza/BCG-infected versus Post-influenza/BCG-infected*)*, 2,140 SDE genes were identified, with the ISG *IFI27* the most significant (Supplementary Data 13). Nine gene clusters were identified (Supplementary Fig. 2 and Supplementary Data 13), of which two were associated with responses to BCG *lux* infection (Clusters 1 and 2, Fig. 3c). Cluster 1 genes (*n* = 67) demonstrated responsiveness to BCG *lux* infection in the pre-influenza samples. In the post-influenza samples, baseline expression was much higher, but there was no discernible response to BCG *lux*. Cluster 1 contained multiple ISGs, including *LY6E, RSAD2, IFI44, IFI44L, OAS1, OAS2, OAS3, OASL, IFIT1, IFIT2, IFIT3, IFIT5, IFITM1, MX1* and *IRF7*. Pathway analysis in IPA revealed overlapping significant pathways with those from the DESeq2 analysis, with the most significant including Role of Hypercytokinaemia/hyperchemokinaemia in the Pathogenesis of Influenza, Interferon Signalling, Activation of IRF by Cytosolic Pattern Recognition Receptors and Role of Pattern Recognition Receptors in Recognition of Bacteria and Viruses (Supplementary Data 14). Cluster 2 (104 genes) included the genes *CASP7, CCL22, CCL8, CCL4, CXCL2, CCL3, IL12B, TNF* and *TICAM1* and was enriched for multiple immunological pathways, including Pathogen Induced Cytokine Storm Signalling Pathway and Role of Hypercytokinaemia/hyperchemokinaemia in the Pathogenesis of Influenza (Supplementary Data 15). Overall, the trend for Cluster 2 genes was higher baseline and peak expression levels in the post-influenza versus pre-influenza samples. Significant pathways were also identified with IPA for Clusters 3, 4, 5, 6 and 8 (Supplementary Data 16–20).

### Relationship between hub gene expression and post-influenza reduction of mycobacterial growth restriction

Most but not all influenza PCR+ participants showed reduced BCG *lux* growth restriction following influenza infection. We hypothesised there would be differences in behaviour of key BCG: Influenza interaction genes, between those with and without reduced BCG *lux* growth restriction following influenza infection (i.e., Non-restrictors versus Restrictors defined as those with ΔGR_72 h_ > 100% and < 100% respectively). Firstly, we identified hub genes, by predicting a protein-protein interaction (PPI) network from the 63 DESeq2 BCG: Influenza interaction genes with the Search Tool for the Retrieval of Interacting Genes (STRING) database in Netscape and then used cytoHubba^41^ to identify the top 10 most highly interconnected genes, which were all ISGs (*RSAD2*, *MX1*, *OAS1, OAS2, OAS3, IFI6*, *IRF7, IFI35, BST2* and *IFI44L*). Then we compared baseline (timepoint 0 h) expression of these hub genes between Restrictors versus Non-restrictors, as well as the change in expression (Δ-expression) of these genes in response to BCG *lux* infection with respect to baseline expression.

**Fig. 4:**
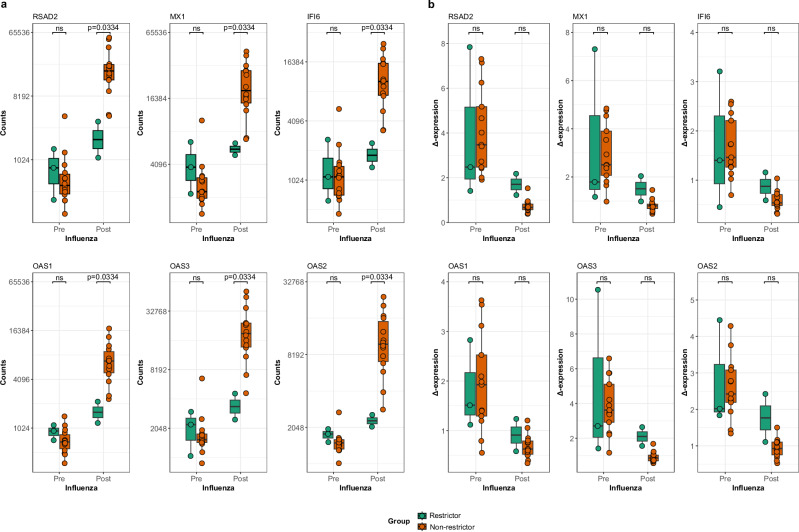
Hub gene responses to BCG *lux* differ in growth restrictors versus non-restrictors. **a** Baseline expression of the top six hub genes in Non-restrictors and Restrictors. Box and whiskers plots of gene expression at the 0 h timepoint for influenza PCR + participants, pre- and post-influenza infection, in Non-restrictors (orange) and Restrictors (green). The boxes represent the interquartile range (IQR), horizontal lines show the median counts, whiskers denote adjacent values within 1.5 IQR of the first and third quartiles. Two-tailed Wilcoxon rank sum was used for comparisons of median expression between Non-restrictors versus Restrictors in both the pre- and post-influenza samples. Non-restrictors pre-influenza *n* = 14, post-influenza *n* = 14; Restrictors pre-influenza *n* = 3, post-influenza *n* = 2. **b** Change in expression of the top six hub genes. Box and whiskers plots for the top six hub genes showing the Change in expression at 6 h (Δ-expression), calculated as: Expression at 6 h in BCG *lux*-infected samples / expression at 0 h in BCG *lux*-uninfected samples. The boxes represent the IQR, the horizontal lines show the median Δ-expression, and whiskers denote adjacent values within 1.5 IQR of the first and third quartiles. Two-tailed Wilcoxon rank sum was used for comparisons of median Δ-expression between Non-restrictors (orange) versus Restrictors (green) in both the pre- and post-influenza samples. Non-restrictors pre-influenza *n* = 13, post-influenza *n* = 14; Restrictors pre-influenza *n* = 3, post-influenza *n* = 2. In all plots, false discovery rate (FDR)-corrected *p*-values are shown; ns denotes adj. *p* > 0.05.

Focusing on the top six hub genes (*RSAD2*, *MX1*, *IFI6*, *OAS1*, *OAS3* and *OAS2)*: in the pre-influenza samples, there were no significant differences between Restrictors and Non-restrictors in either baseline expression or Δ-expression. However, in the post-influenza samples, Non-restrictors demonstrated significantly higher baseline expression of these top six genes and a trend towards reduced responsiveness of these genes to BCG *lux* infection compared to Restrictors, although the threshold of significance was not reached (Fig. 4).

### Reduced interferon production in response to mycobacteria following influenza infection

Having observed that influenza infection activated multiple ISGs and immune response genes, but reduced their subsequent responses to mycobacterial stimulation, we hypothesised that a similar pattern would be seen with type 1 IFN cytokines. We measured IFN-α2a and IFN-β in the MGIA’s supernatants as well as other cytokines implicated in TB pathogenesis previously^42^ (IFN-γ, TNFα, interleukin (IL)-1β, IL-10, IL-17A/F, IL-22 and IL-23). Despite using an ultrasensitive assay, IFN-β levels in the supernatants were too low to accurately quantify.

**Fig. 5:**
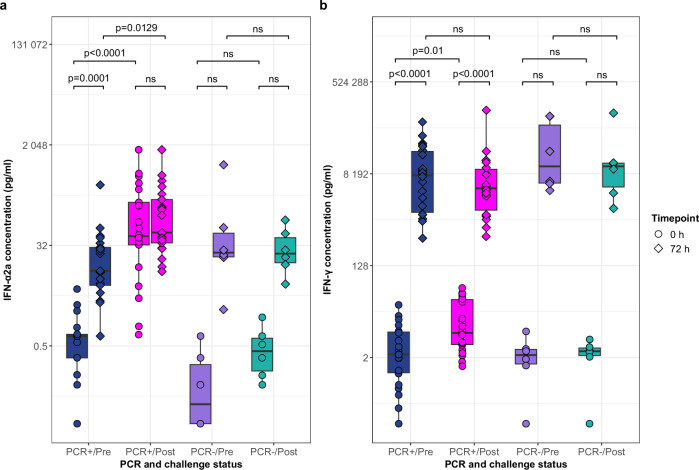
Interferon responses to BCG *lux* infection, pre- and post-influenza. Concentrations of cytokines were measured in the supernatants from BCG *lux*-uninfected blood at baseline (0 h incubation) and BCG *lux*-infected blood at 6, 24 and 72 h incubation. Plots showing baseline (circles) and maximal (diamonds) concentrations of (**a**) IFN-α2a and (**b**) IFN-γ. Box and whiskers plots are shown for 22 influenza PCR + (pre-influenza [blue], post-influenza [pink]) and 6 PCR- (pre-influenza [purple], post-influenza [green]) participants, with the boxes representing the interquartile ranges (IQR), the horizontal lines showing the medians and whiskers denoting adjacent values within 1.5 IQR of the first and third quartiles. Paired comparisons of medians were made using two-tailed Wilcoxon rank sum between the pre- versus post-influenza samples, and baseline versus maximal concentrations, in the PCR + and PCR- groups. In all plots, false discovery rate (FDR)-corrected *p*-values are shown; ns denotes adj. *p* > 0.05. Source data are provided as a Source Data file.

In the pre-influenza blood samples, concentrations of all measured cytokines increased after incubation with BCG *lux* (Fig. 5 and Supplementary Fig. 3), though the threshold of significance was not always reached in the PCR- cohort which had a modest sample size.

Following influenza infection of the PCR+ cohort, baseline IFN-α2a concentration was significantly higher compared to pre-influenza levels, and there was no significant increase in IFN-α2a following incubation with BCG *lux* (Fig. 5a), consistent with the trends we had observed in the RNA expression data. In contrast, IFN-α2a expression patterns in the PCR- cohort were similar in the pre- and post-influenza blood samples.

IFN-γ concentration increased significantly in response to BCG *lux* both before and after influenza infection in the PCR+ participants. While baseline IFN-γ concentration was significantly higher following influenza infection, there was no significant difference in peak concentration after 72 h incubation with BCG *lux* between the pre- versus post-influenza samples, suggesting influenza infection may have diminished the magnitude of the response to BCG *lux* (Fig. 5b). In the PCR- participants, IFN-γ production in response to BCG *lux* appeared similar between the pre- and post-influenza samples. IL-10 followed a similar pattern to IFN-γ (Supplementary Fig. 3a).

Following influenza infection, baseline and peak TNF-α concentrations were significantly higher compared to pre-influenza, as was peak but not baseline IL-1 β concentration (Supplementary Fig. 3b, c). No significant differences in IL-17A/F, IL-22 or IL-23 were observed between the pre-and post-influenza samples, at baseline or peak (Supplementary Fig. 3d–f).

### Altered cellular responses to mycobacteria following influenza infection

**Fig. 6:**
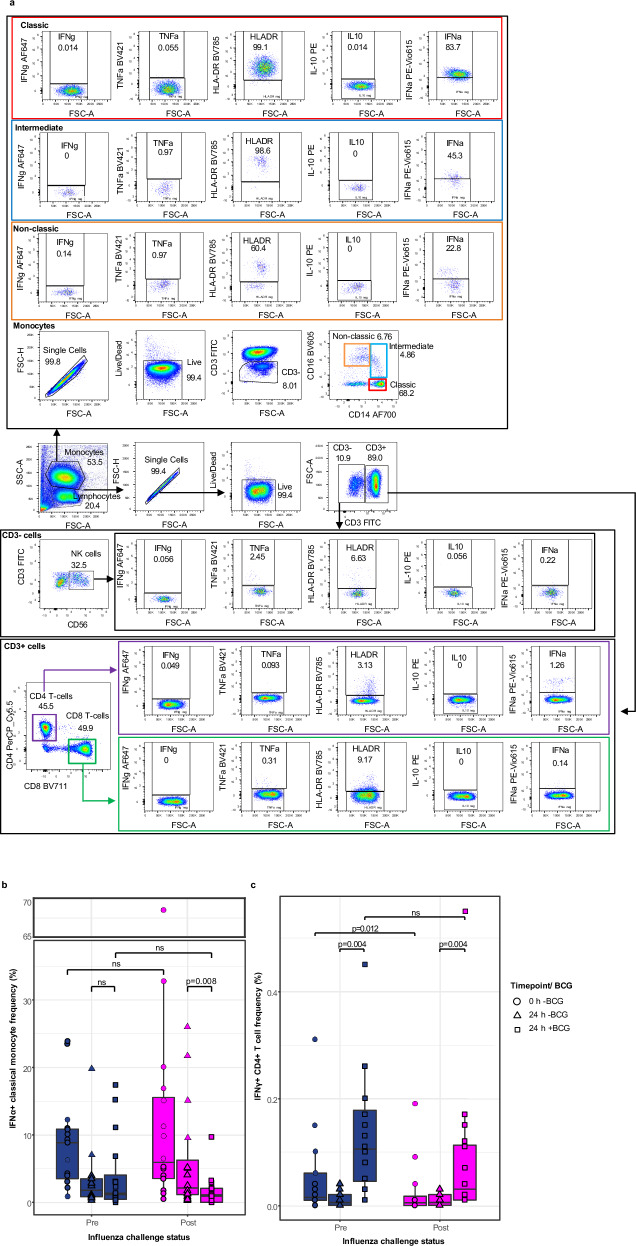
Changing frequencies of interferon-producing cell subsets over time. **a** Gating strategy. Representative dot plots shown. Cells were gated using forward versus side scatter (FSC-A/SSC-A), with dead cells and doublets excluded based on viability staining and forward scatter height versus forward scatter area (FSC-H/FSC-A). Total lymphocytes were identified as CD3 + and further classified into CD4 + helper and CD8 + cytotoxic T cells. NK cells were defined by CD3-CD56 + expression. Total monocytes were identified as CD3-CD14 + cells, with subsets distinguished by CD14 and CD16 expression: classical (CD14 + + CD16-), intermediate (CD14 + + CD16 + +), and non-classical (CD14 + CD16 + +). Cytokine expression (IFN-α, IFN-γ, TNF-α and IL-10) and HLA-DR expression were assessed across all cell subsets. Box and whiskers plots showing the frequency of (**b**) IFN-α + classical monocytes and (**c**) IFN-γ CD4 + T cells over time in 16 influenza PCR + participants. Cell subset frequency in BCG *lux*-uninfected blood at baseline (0 h, circles) and BCG *lux*-uninfected and infected blood at 24 h (triangles and squares respectively) are shown, before (blue) and after (pink) influenza infection, with the boxes representing the interquartile range (IQR), the horizontal lines showing the medians and whiskers denoting adjacent values within 1.5 IQR of the first and third quartiles. Paired comparisons of medians were made using two-tailed Wilcoxon rank sum between pre- versus post-influenza samples, and BCG *lux*-infected versus -uninfected samples. In plots (**b**, **c**), false discovery rate (FDR)-corrected *p*-values are shown; ns denotes *p* > 0.05. Source data are provided as a Source Data file.

In order to confirm the observed transcriptomic and cytokine responses to coinfection, we designed flow cytometry panels to target key cell populations anticipated to produce the cytokines of interest (IFN-α, IFN-γ, TNF-α and IL-10), using cells acquired and stored from blood aliquots used in the MGIA from 16 influenza PCR+ participants. BCG *lux*-uninfected blood aliquots from baseline (timepoint 0 h) as well as paired BCG *lux*-infected and uninfected samples from timepoints 6 h and 24 h were analysed (Fig. 6a).

Classical monocytes (CD3-CD14 + + CD16-, CM) were the predominant monocyte population. Influenza infection did not significantly alter the frequency of IFNα + CM in the baseline samples. IFNα + CM frequency decreased with incubation over time in both BCG *lux*-infected and uninfected samples. Following influenza infection, IFNα + CM frequency was significantly lower in samples incubated with BCG *lux* versus those incubated without BCG *lux*, a difference which was not seen in the pre-influenza samples (Fig. 6b). Similar effects of BCG *lux*-influenza coinfection were seen in other IFNα+ monocyte subsets (Supplementary Fig. 4a, b) as well as IFNα + NK lymphocytes (CD3-CD56 + ) (Supplementary Fig. 4c).

IFN-γ + CD4 + T cell frequency increased in response to BCG *lux* infection in both pre- and post-influenza samples. After 24 h incubation with BCG *lux*, IFN-γ + CD4 + T cell frequency appeared lower in the post-influenza versus pre-influenza samples, though the threshold of significance was not reached (adj *p* = 0.08, Fig. 6c).

TNF-α + CM frequency increased significantly in response to BCG *lux* infection in both the pre- and post-influenza samples. Influenza infection did not alter TNF-α + CM frequency at baseline, but after 6 h incubation with BCG *lux*, there was a trend towards higher TNF-α + CM frequency in the post-influenza versus pre-influenza samples (adj *p* = 0.052, Supplementary Fig. 4d). Influenza infection did not alter IL-10 + CM frequency at baseline, but after 24 h incubation with BCG *lux*, frequency was significantly lower in the post-influenza versus pre-influenza samples (Supplementary Fig. 4e).

## Discussion

We utilised a functional whole blood mycobacterial growth inhibition assay within a human influenza challenge model to investigate the impact of systemic influenza infection on susceptiblity to mycobacteria. We found that influenza infection impairs whole blood control of mycobacterial growth, reducing growth inhibition. Our findings are consistent with published animal model data^10–13^ and epidemiological and observational studies^8,9,14^, including those reporting temporal associations between seasonal influenza and TB^5^, a correlation between seasonal influenza vaccine and reduced TB incidence^6^, increased progression to TB disease in those with upper respiratory tract viruses^15^, including influenza^43^ and higher mycobacterial loads in patients with influenza coinfection at the time of TB diagnosis^44^. Our study is the first to provide convincing evidence for a TB-influenza interaction in humans, with insights into the underlying mechanisms.

In this study, we observed that prior activation of innate immunological pathways in response to influenza, including type 1 IFN signalling, limited their subsequent ability to respond to mycobacterial infection. The differences in expression patterns of the IFN-stimulated hub genes in Restrictors versus Non-restrictors suggest this may be a key mechanism underlying the observed reduction in mycobacterial control following influenza infection. This is in keeping with a murine model of TB-influenza coinfection that reported enhanced mycobacterial growth following influenza infection and a role for type 1 IFN signalling^10^. The detrimental effects of virus-driven immune activation on TB immunity have also been suggested by studies of CMV and *Mtb* infection^45,46^. The role of type 1 IFN responses in TB pathogenesis has been extensively studied^42,47^. Elevated type 1 IFN gene signatures are one of the earliest perturbations in the whole blood transcriptome in individuals progressing from TB infection to disease^34,36^ and are reversable upon successful treatment^48^. Prior studies, in particular mouse models, have suggested a detrimental role for type 1 IFN responses, increasing susceptibility to mycobacterial infection^10,47,49,50^. However, recent non-murine experimental studies support a protective role. In macaques receiving BCG, an early type 1 IFN-inducible gene signature predicted protection from subsequent *Mtb* challenge^51^. A zebrafish model observed increased burden and dissemination of *Mycobacterium marinarum* infection following inhibition of type 1 IFN signalling^52^. The addition of IFN-β into a 3-dimensional in vitro experimental model of *Mtb*-infected human mononuclear cells increased mycobacterial growth restriction^53^. In vivo, a study of tuberculin skin test transcriptomes in patients with pulmonary TB found reduced type 1 IFN activity associated with increased severity of radiographic disease^52^. Where type 1 IFNs have been used therapeutically in humans, reports are mixed. Small studies of adjunct IFN-α therapy for TB disease, in conjunction with anti-tuberculous antibiotics, have reported improved outcomes, including reduced mycobacterial burden^54,55^, but its use as an anti-viral against hepatitis C has been linked to TB reactivation^56,57^. Our study provides evidence that it is the context and timing of these type 1 IFN responses that is key in determining their impact on TB immunity. Our data suggest that increased background type 1 IFN stimulation at rest is deleterious, due to repression of subsequent type 1 IFN responses to mycobacteria, which themselves are beneficial in mycobacterial control, as shown by the differences in ISG expression between Restrictors and Non-restrictors.

We also observed reduced IFN-γ and IL-10 responses to BCG *lux* stimulation following influenza coinfection, consistent with published murine models of mycobacterial-viral coinfection^12,13^. A study of *Mtb*- lymphocytic choriomeningitis virus (LCMV) suggested that viral-induced sustained type 1 IFN signalling inhibited *Mtb*-specific IFN-γ-production and impaired pulmonary migration of *Mtb*-specific CD4 + T cells, resulting in higher bacterial burdens and increased disease severity^13^. They noted higher TNF-α production in the coinfected mice, as we observed here. In vivo in children, defective host responses to both specific antigens and non-specific immune stimuli have been observed after both bacterial and viral infection^58^.

The 63 genes we identified as significantly altered by the interaction of influenza and BCG *lux* include those with roles in a variety of immune processes affecting macrophages, B and T cells and NK cells, as well as non-coding genes, which are known to regulate expression of multiple downstream genes^59^. This suggests complexity to the molecular mechanisms underpinning the functional deficits caused by influenza coinfection, which need to be explored further in future studies.

Blood transcriptomic signatures for diagnosing and/ or predicting TB disease commonly include ISGs^36,60,61^, many of which are also perturbed during influenza infection. This may limit their ability to discriminate between influenza and TB^36^. Our data highlight the importance of considering these overlapping transcriptomic features in response to *Mtb* and influenza and their interactions when developing host gene signature biomarkers for TB, so they can be effectively deployed in real world settings where TB-influenza coinfection is likely to be common. An example of this is the Singhania 20-gene signature, specifically designed to discriminate between TB and influenza^36^, which does not include any of the BCG: Influenza interaction genes from our study.

The human influenza challenge model provides a unique opportunity to study the effects of systemic influenza infection on anti-mycobacterial immunity in a highly controlled environment. The ability to make comparisons within the same individuals in a constant environment before and after influenza challenge minimises potential confounders and facilitates the study of complex interactions between co-pathogens and host response. The participants who did not become infected with influenza and remained PCR-negative did not demonstrate increased mycobacterial growth in their blood after influenza inoculation, strongly suggesting the effects described here were due to influenza infection rather than the experimental procedures or another unmeasured confounder, though we recognise the number of PCR- participants was modest. Our study has limitations. The number of participants is modest and resulted in small sample sizes for the comparison of Restrictors versus Non-restrictors. Whilst we observed significant differences in some hub gene responses between Restrictors and Non-restrictors, for other hub genes, the observed trends were non-significant. These comparisons may have been subject to a type 2 error resulting from the small sample size. Our study utilised an in vitro BCG model, which, while well-validated and shown to reflect immune responses in TB, provides a surrogate measure of TB susceptibility, and findings may have differed if a virulent or clinical *Mtb* strain had been used. The clinical impact of influenza infection on outcomes after TB exposure and infection cannot be directly determined from these data, although they do, supported by the pre-existing literature, suggest a deleterious effect of co-influenza infection on in vivo TB infection. Studying the direct impact of influenza on in vivo TB infection requires very large longitudinal cohorts, prolonged follow-up periods and consideration of a multitude of confounders. Thus in vitro studies that utilise surrogates of TB susceptibility and control are invaluable. Transcriptomic responses to in vitro mycobacterial infection have been shown to be similar to those of in vivo infection^29^, but we recognise that TB is primarily a respiratory pathogen and therefore the blood model is unlikely to fully reflect immunological interactions at the site of primary infection, though it is advantageous with respect to safety, cost and acceptability. Coinfection could be explored further using human mycobacterial challenge studies^62–64^. In particular, lung challenge studies, where BCG is instilled directly into the lungs of healthy volunteers via aerosolisation and inhalation^63^ or bronchoscopy^64^, would provide an excellent opportunity to study the interaction of these two respiratory pathogens within the lung compartment.

Understanding the duration of the observed influenza effect on anti-mycobacterial immunity will be important for predicting its potential clinical impact and could be explored using post-influenza convalescent blood samples, which were unavailable for this study. Previous studies suggest influenza infection may have a longer-term effect on mycobacterial immunity. In the case of bacterial pneumonia, which is a well-recognised complication of influenza infection, the increased incidence following influenza is highest in the first week after influenza infection but persists for as long as 30 days^65^. A murine *Mtb*-influenza coinfection model has suggested acute influenza infection has long-term consequences for immunological control of *Mtb*^10^.

This work demonstrates the immunomodulatory effect of a common co-pathogen on anti-mycobacterial immune responses, a better understanding of which is essential for the development of effective vaccinations and for the interpretation of host immune response-based diagnostics for TB. The immune mechanisms noted to be important to the coinfection interaction, including type 1 IFN signalling, are common to many viruses, so it seems likely that this effect of reducing mycobacterial containment will be a broader viral phenomenon rather than specific to influenza. Further knowledge will help us understand, and potentially mitigate for, the impacts of future viral epidemics and pandemics on TB control.

Our findings, together with those from prior epidemiological studies, strongly suggest that influenza infection is a risk factor for TB. Thus, pharmacological and non-pharmacological interventions that reduce influenza transmission may also benefit TB control. In particular, these data support the case for an interventional trial to evaluate the impact of seasonal influenza vaccination on TB incidence.

In summary, we have shown that systemic influenza infection reduces whole blood containment of mycobacteria via its disruption of innate immunological pathways important in anti-mycobacterial control, including type 1 interferon signalling. Our data suggest that influenza infection could be a risk factor for progression of TB disease and that interventions that protect against influenza, such as the seasonal influenza vaccine, could also be protective against TB.

## Methods

### Ethics and consent

The procedures and protocols described here were approved by the Brighton & Sussex Research Ethics Committee, London (reference 19/LO/0208) and London- Fulham Research Ethics Committee (references 11/LO/1826 and 19/LO/1441). Prior to inclusion in the study, written informed consent was obtained from all participants.

### Biosafety statement

Participants were inoculated with influenza virus in an allocated isolation bay or negative pressure room and then quarantined in a confinement unit at the Imperial Clinical Research Facility, Imperial College London, for 10 days post-inoculation if they developed PCR-confirmed infection or 8 days if not. During this time, they were not permitted to exit the unit or receive visitors. Study and facility staff wore personal protective equipment when in the quarantine unit according to local standard operating procedures.

All work on BCG *lux* was performed in Biosafety Level 2 laboratories at Imperial College London according to protocols reviewed and approved by Imperial College London’s Safety Department Bioteam (Activity ID GMIC 01147-1 v 5). All personnel working with mycobacteria were trained with relevant safety and protocol-specific procedures.

### Influenza challenge model

The Influenza A H3N2 human challenge model has been described^66^. Healthy persons aged 18 to 55 years were eligible. Thirty healthy volunteers were inoculated intranasally with Influenza A/Belgium/4217/2015 (H3N2) at a dose of 5 × 10^5^ 50% tissue culture infectious dose (TCID_50_) in a volume of 0.5 mL by drops divided between nostrils, under quarantine conditions. From 24 h after inoculation, the virus was quantified by quantitative PCR in nasal lavage samples obtained at 12 hourly intervals. Participants were ascertained to have replicative viral infection if consecutive positive PCR tests were obtained at least 24 h after the inoculation. Blood samples for the MGIA were collected in lithium heparin tubes (BD, NJ, USA) on day 0 (approximately one hour prior to viral inoculation) and day +6 after virus challenge. Age, and self-reported sex and ethnicity data were collected for each participant. Participants were combined from two sequential challenge studies with identical inclusion/exclusion criteria and processes: ClinicalTrials.gov IDs NCT02755948 and NCT04204993. Study NCT04204993 has published its primary outcomes^66^; study NCT02755948 has not yet published primary outcomes relating to H3N2 influenza. The data reported here are not related to these primary outcomes. As per its protocol, NCT02755948 collected bronchoalveolar lavage samples from participants, but these did not contribute to this study.

### Reporter Mycobacteria bovis BCG lux

The construction and properties of BCG *lux* have been described^20,22^. Briefly, *Mycobacterium bovis* BCG (Montreal strain) was transformed with the plasmid shuttle vector pSMT1, carrying the luciferase *luxAB* genes and a gene encoding hygromycin resistance. The recombinant mycobacteria were grown into the logarithmic phase in Middlebrook 7H9 culture medium (Difco^TM^, BD), supplemented with 50 µg/ml hygromycin, 10% Albumin Dextrose Catalase (ADC) supplement (BD), 15% glycerol and 0.05% Tween 80, in an orbital shaker incubator (Innova 4300, New Brunswick Scientific, Edison, NJ, USA) at 220 RPM and 37 °C. The culture was dispened into 1.2 ml aliquots and frozen at − 80 °C.

Prior to each MGIA assay, a single BCG *lux* aliquot from the same original stock was defrosted and added to 30 ml 7H9 Middlebrook medium, 80 µl 20% Tween 80 and 30 µl 50 mg/ml hygromycin B in a vented Erlenmeyer flask and incubated in an orbital shaker incubator at 220 RPM and 37 °C. The BCG *lux* aliquot was grown to mid-logarithmic phase, and diluted in phosphate-buffered saline (PBS) (Sigma-Aldrich, St Louis, USA) to a final concentration of 2.3 × 10^6^ colony forming units (cfu)/ml.

Luminescence was measured in duplicate samples, diluted 1:10 in PBS to a total volume of 1 ml, over a 20 s period after injection of 0.1 mL of substrate (1% N-decyl aldehyde; Sigma-Aldrich) in a tube luminometer (Berthold AutoLumat Plus, Berthold Technologies, Germany). Results were expressed as relative light units per millilitre (RLU/ml). Enumeration of cfu of mycobacteria was determined by culturing BCG *lux* on Middlebrook 7H11 agar (BBL^TM^, BD) containing 10% Oleic acid Albumin Dextrose Catalase (OADC) supplement (BD) and hygromycin (50 mg/mL). A reading of 4.5 RLU/ml corresponded to 1 cfu.

### Whole blood assay

The WBA was set up within 4 h of blood sample collection from the challenge participants. Fresh whole blood was diluted 1:1 with Roswell Park Memorial Institute (RPMI) 1640/ 2 mM glutamine medium (GibcoTM, Thermo Fisher Scientific). From each diluted blood sample, multiple aliquots were made for parallel experiments to quantify mycobacterial growth inhibition, gene expression, cytokines and cell subsets (Fig. 1).

Where blood aliquots were infected with BCG *lux*, BCG *lux* was added at a 1:10 concentration (final BCG *lux* inoculum 2.3 × 10^5^ cfu/ml) to give a multiplicity of infection of 1:1 (cfu: monocyte), based on an average monocyte count of 2-4 × 10^5^ monocytes /ml whole blood (i.e., 1- 2 × 10^5^ monocytes /ml diluted blood).

### Mycobacterial growth inhibition assay

The BCG *lux* MGIA has been reported^21,22,24^. From the original diluted blood sample, two sets of triplicate blood aliquots (450 µl) were inoculated with 50 µl BCG *lux* and incubated at 37 °C in a rocking incubator (Platform rocker incubator S180, Stuart) at 20 RPM. After 20 min (for timepoint 0 h) and 72 h respectively, a set of triplicates was removed from the incubator and centrifuged for 10 min at 2000 × *g* force (x *g*). Supernatants were removed and filtered using 0.2 µM/ 2 ml cellulose acetate centrifuge tube filters (CostarTM, Corning Inc, USA) and stored in 2 ml screw top polypropylene microtubes (Sarstedt, Nümbrecht, Germany) at − 80 °C. Filtration was a safety requirement to ensure effective removal of mycobacteria prior to multiplex cytokine studies. The remaining cell pellets were each resuspended in PBS, 5 ml distilled water added and then incubated at room temperature for 10 min for red cell lysis. The tubes were then spun for 10 min at 2000 x *g* and the supernatants discarded. Next, 500 µl PBS and 5 glass beads (2 mm diameter, Merck, Darmstadt, Germany) were added and the tubes vortexed to disperse the cell pellets. Luminescence of each triplicate sample was measured in duplicate as described previously, to give six luminescence values per timepoint per participant, from which a median was calculated. Aliquots of the BCG *lux* inoculum were incubated in the same rocker incubator and luminescence measured at the same timepoints, to ensure it grew as expected. An aliquot of the inoculum was also cultured on solid 7H11 agar to confirm the inoculum’s mycobacterial cfu count.

### Transcriptional profiling

From the original diluted blood sample, five 2000 µl aliquots were used for RNA studies: two aliquots were infected with 222 µl BCG *lux,* and the same volume of PBS was added to the remaining three aliquots as uninfected controls. All samples were incubated in the same rocking incubator described above. One uninfected sample was incubated for 20 min (timepoint 0 h), and paired infected and uninfected samples were incubated for 6 h and 24 h. At the appropriate timepoint, aliquots were removed from the incubator and centrifuged at 2000 x *g* for 10 min. The supernatant was removed, filtered as described above and frozen in 2 ml screw top polypropylene microtubes at − 80 °C for future cytokine analysis. To the cell pellet, RNA preservative from a PAXgene® tube (PreAnalytix, GmBH, Switzerland) was added at a ratio of 2.76: 1 undiluted whole blood, as recommended by the manufacturer. The sample was vortexed to disperse the cell pellet and then frozen at − 20 °C overnight before being transferred to − 80 °C for longer-term storage.

RNA was extracted using the PAXgene® blood miRNA kit (PreAnalytix) according to the manufacturer’s manual extraction protocol, with on-column DNase treatment and clean-up using RNA Clean & Concentrator-5 kit (Zymo Research, CA, USA).

Material was quantified using RiboGreen (Invitrogen, MA, USA) on the FLUOstar OPTIMA plate reader (BMG Labtech, Ortenberg, Germany) and the size profile and integrity analysed on the 2200 TapeStation (Agilent). The input material was normalised, and strand-specific library preparation was completed using NEBNext® Ultra™ II mRNA kit (New England BioLabs [NEB], MA, USA) and NEB human v2 rRNA/globin depletion probes (NEB) following the manufacturer’s instructions. Libraries were on a Tetrad (Bio-Rad) using in-house unique dual indexing primers (based on ref. ^67^). Individual libraries were normalised using Qubit (Invitrogen, MA, USA) and pooled together. The pooled library was diluted to ~ 10 nM. Paired end sequencing was performed at The Wellcome Centre for Human Genetics, Oxford, UK using a Novaseq6000 platform (Illumina, CA, USA) at 150 paired end configuration.

Quality control was performed using FastQC^68^ and MultiQC^69^ and annotations were modified with BEDTools^70^. Alignment against the hg38 reference genome version 89 was undertaken using STAR^71^ and SAMtools^72^, followed by transcript quantification using featureCounts^73^. Transcript-level output counts were normalised in DESeq2^30^ (V1.30.1) using the median ratio method (sizeFactors) and were annotated with gene name using biomaRt^74^ (V2.46.3) in R^75^ (V4.0.4). Lowly expressed genes (total counts < 10 and genes for which fewer than three samples had a read count of ≥ 20) and ribosomal genes were filtered out. Principal component analysis (PCA) was performed on the normalised counts for quality control and visualised with ggplot2 (V3.5.1), and confirmed there were no outlying samples.

### Quantification of cytokines

Meso Scale Diagnostics (MSD) U-plex assays (MSD LLC, Maryland, USA) were used to quantify IFN-γ, TNF-α, IL-1β, IL-10, IL-17A/F, IL-22 and IL-23 in supernatants following the manufacturer’s instructions, and MSD S-plex assays to quantify IFN-α2a and IFN-β (Supplementary Table 3). Plates were read using MSD 1300 Meso QuickPlex with MSD Methodical Mind software. MSD discovery workbench 4.0 software was utilised to plot and quality check standard curves and calculate analyte concentrations.

### Flow cytometry

From each diluted blood sample, five 390 µl aliquots of diluted blood were prepared for flow cytometry studies: two aliquots were infected with 43 µl BCG *lux* and three uninfected controls with 43 µl PBS added. One uninfected sample was incubated for 20 min (timepoint 0 h), and paired infected and uninfected samples were incubated for 6 h and 24 h. Brefeldin A was added for the final 4 h of the incubation.

On removal from the incubator, 9 µl of 0.1 M liquid EDTA and 8.5 ml alternative lysis solution (ammonium chloride lysing solution^76^) were added, the sample vortexed and incubated at room temperature for 10 min. After centrifugation at 500 x *g* for 5 min, the supernatant was poured off, the cells washed in PBS, and 100 µl of 1% Zombie NIR Fixable Viability kit (BioLegend, San Diego, CA, USA) then added, followed by a 20 min incubation in the dark at 4 C°. After washing the cells, they were fixed with paraformaldehyde and cryopreserved (using 1 ml 10% dimethyl sulfoxide (DMSO) and 25% heat inactivated foetal calf serum (FCS) in RPMI with L-glutamine) before storage at − 80 °C.

Cryopreserved cells were thawed, washed, and permeabilized with Perm/wash solution (BD Biosciences, San Jose, CA, USA). Cells were then incubated at 4 °C for 1 h with fluorescence-conjugated antibodies directed against surface antigens and intracellular cytokines (Supplementary Table 3). Cells were washed and resuspended in FACS buffer (PBS, 2% FCS, 2 µM EDTA) for acquisition.

The entire sample was acquired on a BD LSR Fortessa Flow Cell Analyser (BD Biosciences) using FACSDiva software (version 9.2, BD Biosciences). Compensation was performed for the overlap of fluorescence detection using compensation beads. Control samples had previously been used to evaluate appropriate staining controls of antibody and fluorochrome interactions and spectral overlap. Data were analysed using FlowJo (version 10.10.0, Ashland, OR, USA).

### Gating strategy

Cells were gated using forward versus side scatter (FSC-A/SSC-A), with dead cells and doublets excluded based on viability staining and forward scatter height versus forward scatter area (FSC-H/FSC-A). Total lymphocytes were identified as CD3 + and further classified into CD4 + helper and CD8 + cytotoxic T cells. NK cells were defined by CD3-CD56 + expression. Total monocytes were identified as CD3-CD14 + cells, with subsets distinguished by CD14 and CD16 expression: classical (CD14 + + CD16-), intermediate (CD14 + + CD16 + +), and non-classical (CD14 + CD16 + +). Cytokine expression (IFN-α, IFN-γ, TNF-α and IL-10) and HLA-DR expression were assessed across cell subsets (Fig. 6A).

### Statistical analyses

Tests of normality were performed using the Shapiro-Wilk’s method. For normally distributed data, comparisons between means were made using *t* tests. For data that were not normally distributed, the Wilcoxon rank sum was utilised for comparisons of medians. All tests were two-tailed, and *p* ≤ 0.05 was considered significant. Where paired data were available, paired analyses were undertaken, and this is indicated in the results.

### Mycobacterial growth inhibition

The average luminescence for each time point was calculated as the median of the six luminescence values measured for that participant’s blood sample. GR_72 h_ was calculated as the median luminescence at 72 h incubation divided by the median luminescence at 0 h. Change in GR_72 h_ (ΔGR_72 h_) was calculated as post-influenza GR_72 h_ / pre-influenza GR_72 h_ expressed as a percentage. BCG *lux* GR_72 h_ and ΔGR_72 h_ were plotted in GraphPad Prism (version 9 for Windows, GraphPad Software, Boston, MA, USA, www.graphpad.com) and comparisons made between pre- versus post- influenza samples (paired), and influenza PCR+ versus PCR- participants (unpaired) using Wilcoxon rank sum.

### Transcriptomic analyses

To focus subsequent differential analyses on genes exhibiting temporal dynamics, a filtering step was added. We used DESeq2^30^ to identify genes significantly associated with Timepoint (0 h, 6 h, 24 h), applying a model design considering only Timepoint ( ~ Timepoint). This analysis was conducted on the full dataset and four key experimental subsets stratified by BCG *lux* infection and influenza status (Pre-influenza/BCG-uninfected; Pre-influenza/BCG-infected; Post-influenza/BCG-uninfected; Post-influenza/BCG-infected). Following Benjamini-Hochberg (BH) adjustment^31^ for false discovery rate (FDR), genes with an adjusted *p*-value (adj. *p*) ≤ 0.05 in any of these five analyses were considered temporally variable. The union of these temporally variable genes formed the final gene set which, following further filtering to remove lowly expressed genes (removing genes for which fewer than 5 samples had a read count of ≥ 30), were used for the main paired DESeq2 and maSigPro differential expression analyses (*n* = 17,940).

Paired differential expression analyses was undertaken in DESeq2 using the default parameters. The model design included the variables Timepoint (0 h, 6 h, 24 h), BCG (BCG *lux* uninfected/ infected), Influenza (pre- and post-influenza infection), the interaction terms BCG: Timepoint, to allow for the differing effects of incubation time depending on the presence or absence of BCG *lux* and the interaction BCG: Influenza to identify genes of interest for our hypothesis. Adjustment for FDR was performed using the BH procedure. Genes with adj. *p* ≤ 0.05 were considered to be SDE. Expression of individual genes was plotted using the package ggplot2. Heatmap visualisation of the BCG: Influenza interaction genes was made using the package pheatmap (V1.0.12).

A second DESeq2 model was constructed, similar to the first but with the addition of in silico estimated total lymphocyte, total monocyte and neutrophil fractions, to enable adjustment for transcriptomic differences induced by differing proportions of immune cells. Estimation of immune cell-type fractions from the bulk host transcriptome data was performed using CIBERSORTx^77^. The following cell fractions were estimated: monocytes, macrophages (the sum of the proportions of macrophages M0, macrophages M1, macrophages M2), dendritic cells (the sum of the proportions of resting and activated dendritic cells), neutrophils, B cells (the sum of the proportions of naïve and memory B cells and plasma cells), CD4 T cells, (the sum of the proportions of naïve CD4 T cells, resting and activated memory CD4 T cells), CD8 T cells, follicular helper T cells, regulatory T cells  and natural killer (NK) cells (the sum of the proportions of resting and activated NK cells). Total monocyte fraction was calculated as the sum of total macrophages, monocytes and dendritic cells. Total lymphocyte fraction was calculated as the sum of total B cells, CD4 + T cells, CD8 + T cells, follicular helper T cells, regulatory T cells and NK cells.

The R package maSigPro^37,38^ (V1.78.0) was utilised to identify genes SDE over time and between the experimental groups: Pre-influenza/BCG-uninfected; Pre-influenza/BCG-infected; Post-influenza/BCG-uninfected; Post-influenza/BCG-infected, with Pre-influenza/BCG-infected specified as the comparator group, to enable direct comparison of Post-influenza/BCG-infected versus Pre-influenza/BCG-infected. maSigPro employs a two-step regression strategy to identify genes with significant expression changes over time and between experimental groups

So as to align with the DESeq2 analysis and to enable comparisons to be made, the same samples were used in the maSigPro analysis as were used in the paired DESeq2 analysis (i.e., timepoints 0 − 24 h). In order for longitudinal comparisons to be made between the four groups described above, expression data were required for timepoint 0 h in all four groups. We assumed that at timepoint 0 h, the variances in the BCG-infected samples would be the same as those in the BCG-uninfected samples, and there would be no differences in expression between BCG-infected and uninfected samples before inoculation with BCG *lux*. We duplicated the expression data from the BCG-uninfected 0 h samples to provide expression data for the BCG-infected 0 h samples for the maSigPro analysis: Timepoint 0 h Pre-influenza/BCG-uninfected was duplicated to give Timepoint 0 h Pre-influenza/BCG-infected; Timepoint 0 h Post-influenza/BCG-uninfected; was duplicated to give Timepoint 0 h Post-influenza/BCG-infected. In maSigPro, two degrees of freedom to capture quadratic trends and binomial distribution were specified and the argument vars = “each” so that SDE genes for all variables in the regression model would be generated. Genes with a BH adj. *p* ≤ 0.05 and *R*^2^ > 0.6 were considered SDE. Coefficients obtained in the maSigPro comparisons were then used to cluster significant genes with similar temporal expression patterns using hierarchical clustering, with maSigPro’s default settings.

Pathway analysis was undertaken using Ingenuity Pathway Analysis (IPA; QIAGEN Inc., https://www.qiagenbioinformatics.com/products/ingenuity-pathway-analysis). Adjustment for FDR was performed using the BH procedure, and pathways with adj. *p* ≤ 0.05 were considered to be significant. For each predicted pathway, a z-score predicting the directionality of the canonical pathways was calculated using the LFC values generated from the differential expression analysis. Core analyses were conducted in IPA using the default settings, with human species specified and “Cell lines” removed from the analysis. IPA recommends an “ideal” gene set size of 200–3000 genes for core analysis. Where differential expression analyses had generated high numbers of SDE genes, filtering strategies based on LFC and adj. *p*-value cut offs were employed to reduce the number of SDE genes for core analysis and are noted where applicable. Cancer pathways were excluded from the canonical pathways results.

Protein-protein interaction (PPI) networks were constructed using the Search Tool for the Retrieval of Interacting Genes (STRING) online database^78^ and the network model visualised using Cytoscape^79^ (V3.9.1). The confidence score threshold was set at 0.9, so that only interactions predicted with the highest confidence level were included in the predicted network. CytoHubba was used to rank the protein nodes in the network, identifying the top ten most interconnected proteins and their encoding genes using Maximal Clique Centrality^41^.

For comparisons of Restrictors versus Non-restrictors, all available RNA sequencing data were utilised, including two participants with individual missing RNA samples that had excluded them from the original paired DESeq2 analysis. This maximised the number of participants in the smaller Non-restrictor group, allowing the inclusion of 14 Restrictors and 3 Non-restrictors. Change in gene expression at 6 h (Δ -expression) was calculated as: Expression at 6 h in BCG *lux*-infected samples / expression at 0 h in BCG *lux*-uninfected samples.

Two-tailed Wilcoxon rank sum was used for comparisons of median expression and Δ-expression, and *p*-values adjusted for FDR with the BH procedure, using the packages dplyr (V1.1.4), tidyr (V1.3.1) and rstatix (V0.7.2) and visualised using ggplot2, ggbreak (V0.1.4), ggsignif (V0.6.4), gridExtra (V2.3) and cowplot (V1.1.3). Where appropriate, paired comparisons were made.

### Cytokine analyses

Analyses were conducted using the statistical software R using the packages dplyr, tidyr and rstatix and visualised using ggplot2, ggbreak, ggsignif, gridExtra, cowplot and scales (V1.4.0). The mean concentration of each duplicate pair was calculated. Undetectable sample values were given the value of half the lower limit of detection. Comparisons of median absolute cytokine concentrations were made using two-tailed Wilcoxon rank sum, with *p*-values adjusted for FDR using the BH procedure. Where appropriate, paired comparisons were made.

### Cell subset analyses

Analyses were conducted using the statistical software R using the packages tidyr and rstatix and visualised using ggplot2, ggbreak, ggsignif, gridExtra, scales and cowplot. Comparisons of median cell frequency were made using two-tailed Wilcoxon rank sum, with *p*-values adjusted for FDR using the BH procedure. Where appropriate, paired comparisons were made.

### Reporting summary

Further information on research design is available in the Nature Portfolio Reporting Summary linked to this article.

## Supplementary information

Supplementary Information

Description of Additional Supplementary Files

Supplementary Data 1

Supplementary Data 2

Supplementary Data 3

Supplementary Data 4

Supplementary Data 5

Supplementary Data 6

Supplementary Data 7

Supplementary Data 8

Supplementary Data 9

Supplementary Data 10

Supplementary Data 11

Supplementary Data 12

Supplementary Data 13

Supplementary Data 14

Supplementary Data 15

Supplementary Data 16

Supplementary Data 17

Supplementary Data 18

Supplementary Data 19

Supplementary Data 20

Reporting summary

Transparent Peer Review file

## Source data

Source Data

## Data Availability

The processed RNA sequencing data and associated metadata generated in this study are available at ArrayExpress under accession code E-MTAB-15137. To comply with data privacy restrictions and the study’s ethics agreement, raw sequencing data are available under managed access through the European Genome-Phenome Archive (https://ega-archive.org), under accession number EGAS50000001677. Data will be shared with investigators whose proposed use is within the scope of participant consent, on request to the corresponding author, subject to a data access agreement. Source data are provided in this paper.

## Code availability

The R code for the whole blood transcriptional profiling analyses can be found at https://github.com/cmbroderick/bcg_influenza. A permanent repository for the code is at 10.5281/zenodo.17880285.
