# Supplementary material for: Influenza coinfection inhibits control of mycobacterial infection in a human challenge model
Source: Nat Commun. 2026 Jun 11;17:4884. doi: 10.1038/s41467-026-72363-2 (PMC13261052; doi:10.1038/s41467-026-72363-2)
Supplement: Supplementary file 1 — Supplementary Information [file 41467_2026_72363_MOESM1_ESM.pdf]

Influenza coinfection inhibits control of mycobacterial infection in a human challenge model

Supplementary Information

Table of Contents

Supplementary Figures 2

Supplementary Figure 1. Schematic overview of whole blood samples used for transcriptomics analyses. 2

Supplementary Figure 2. Nine gene clusters identified by maSigPro. 3

Supplementary Figure 3. Cytokine responses to BCG *lux* infection, pre- and post-influenza. 4

Supplementary Figure 4. Changing frequencies of cell subsets over time. 5

Supplementary Tables 6

Supplementary Table 1. Demographics of influenza challenge patients testing PCR-positive (+) and PCR-negative (-) for Influenza A (H3N2) virus. 6

Supplementary Table 2. Comparisons of baseline (pre-influenza) BCG *lux* $GR_{72\ h}$ and $\Delta GR_{72\ h}$ for demographics and viral load. 7

Supplementary Table 3. Antibodies 8

Supplementary References 8

For Supplementary Data 1-20, please see individual Excel files and "Legends for supplementary data 1-20"

Supplementary Data 1. Genes significantly differentially expressed for the BCG: Influenza interaction and their associations with anti-mycobacterial immune responses.

Supplementary Data 2. Significantly differentially expressed genes identified in DESeq2 for the variable BCG.

Supplementary Data 3. Significantly differentially expressed genes identified in DESeq2 for the variable Influenza.

Supplementary Data 4. Significant pathways identified using IPA for BCG: Influenza.

Supplementary Data 5. Significant pathways identified using IPA for BCG.

Supplementary Data 6. Significant pathways identified using IPA for Influenza.

Supplementary Data 7. Significantly differentially expressed genes identified in DESeq2 with the cell-adjusted model for the BCG: Influenza interaction.

Supplementary Data 8. Significantly differentially expressed genes identified in DESeq2 with the cell-adjusted model for the variable BCG.

Supplementary Data 9. Significantly differentially expressed genes identified in DESeq2 with the cell-adjusted model for the variable Influenza.

Supplementary Data 10. Significant pathways identified using IPA for BCG: Influenza (cell-adjusted model).

Supplementary Data 11. Significant pathways identified using IPA for BCG (cell-adjusted model).

Supplementary Data 12. Significant pathways identified using IPA for Influenza (cell-adjusted model).

Supplementary Data 13. Significantly differentially expressed genes identified by maSigPro.

Supplementary Data 14. Significant pathways identified using IPA for Cluster 1.

Supplementary Data 15. Significant pathways identified using IPA for Cluster 2.

Supplementary Data 16. Significant pathways identified using IPA for Cluster 3.

Supplementary Data 17. Significant pathways identified using IPA for Cluster 4.

Supplementary Data 18. Significant pathways identified using IPA for Cluster 5.

Supplementary Data 19. Significant pathways identified using IPA for Cluster 6.

Supplementary Data 20. Significant pathways identified using IPA for Cluster 8.

Supplementary Figures

Supplementary Figure 1. Schematic overview of whole blood samples used for transcriptomics analyses. Each green and purple box represents a whole blood sample for RNA-sequencing, with each participant contributing 10 unique samples to the model. Created in BioRender. Broderick, C. (2025) https://BioRender.com/jigrub5.

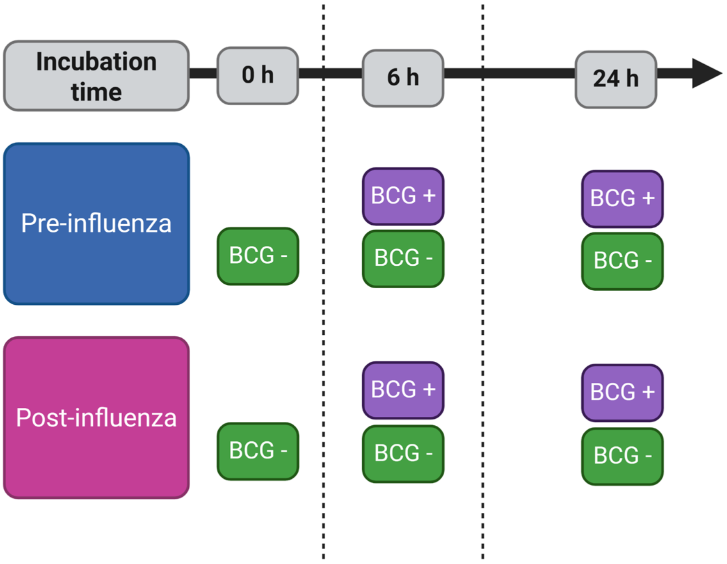

Supplementary Figure 2. Nine gene clusters identified by maSigPro. Analysis of time-course gene expression data was performed using the maSigPro package, which uses a two-step polynomial regression approach. Significance of temporal changes and differences between groups was assessed using an F-test. P-values from this test were adjusted for multiple comparisons using the Benjamini-Hochberg (BH) method. In total, 2,140 genes were identified as significantly differentially expressed (SDE) over time and between groups (BH adj. $p \leq 0.05$, $R^2>0.6$). The coefficients obtained were used to group together SDE genes into clusters with similar temporal expression patterns. Summary plots of gene expression over time for the BCG-infected pre-influenza (dark blue), BCG-infected post-influenza (pink), BCG-uninfected pre-influenza (light blue) and BCG-uninfected post-influenza (red) groups are shown. Solid lines connect the median expression to show the trends for each group, and the dashed lines show the regression curves fitted to the data.

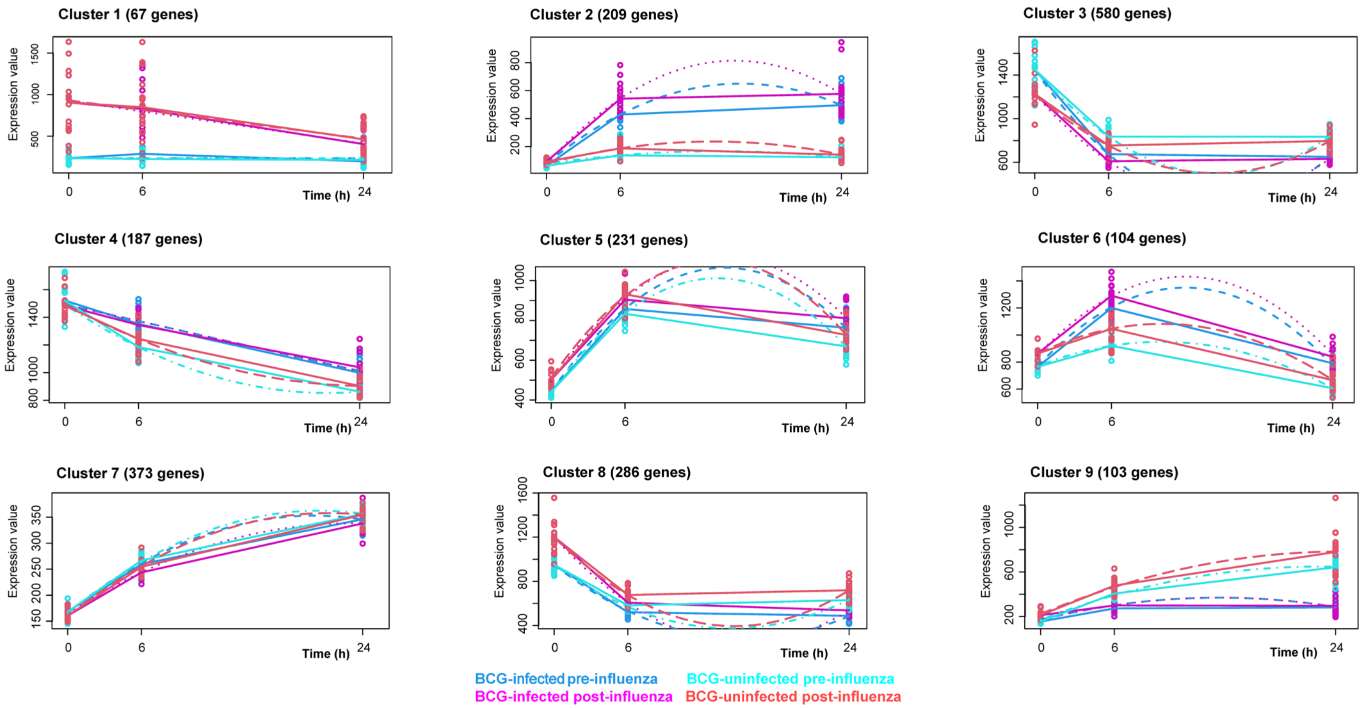

Supplementary Figure 3. Cytokine responses to BCG *lux* infection, pre- and post-influenza. Concentrations of **a** IL-10, **b** TNF-α, **c** IL-1β, **d** IL 17-A/F, **e** IL-22 and **f** IL-23 were measured in the supernatants from BCG *lux*-uninfected blood at baseline (0 h incubation) and BCG *lux*-infected blood at 6, 24 and 72 h incubation. Plots showing baseline (circles) and maximal (diamonds) concentrations. Box and whiskers plots are shown for 22 influenza PCR+ (pre-influenza [blue], post-influenza [pink]) and 6 PCR- (pre-influenza [purple], post-influenza [green]) participants, with the boxes representing the interquartile ranges (IQR), the horizontal lines showing the medians and whiskers denoting adjacent values within 1.5 IQR of the first and third quartiles. Paired comparisons of medians were made using two-tailed Wilcoxon rank sum between the pre- versus post-influenza samples, and baseline versus maximal concentrations, in the PCR+ and PCR- groups. In all plots, false discovery rate (FDR)-corrected p values are shown; ns denotes adj. p >0.05. Source data are provided as a Source Data file.

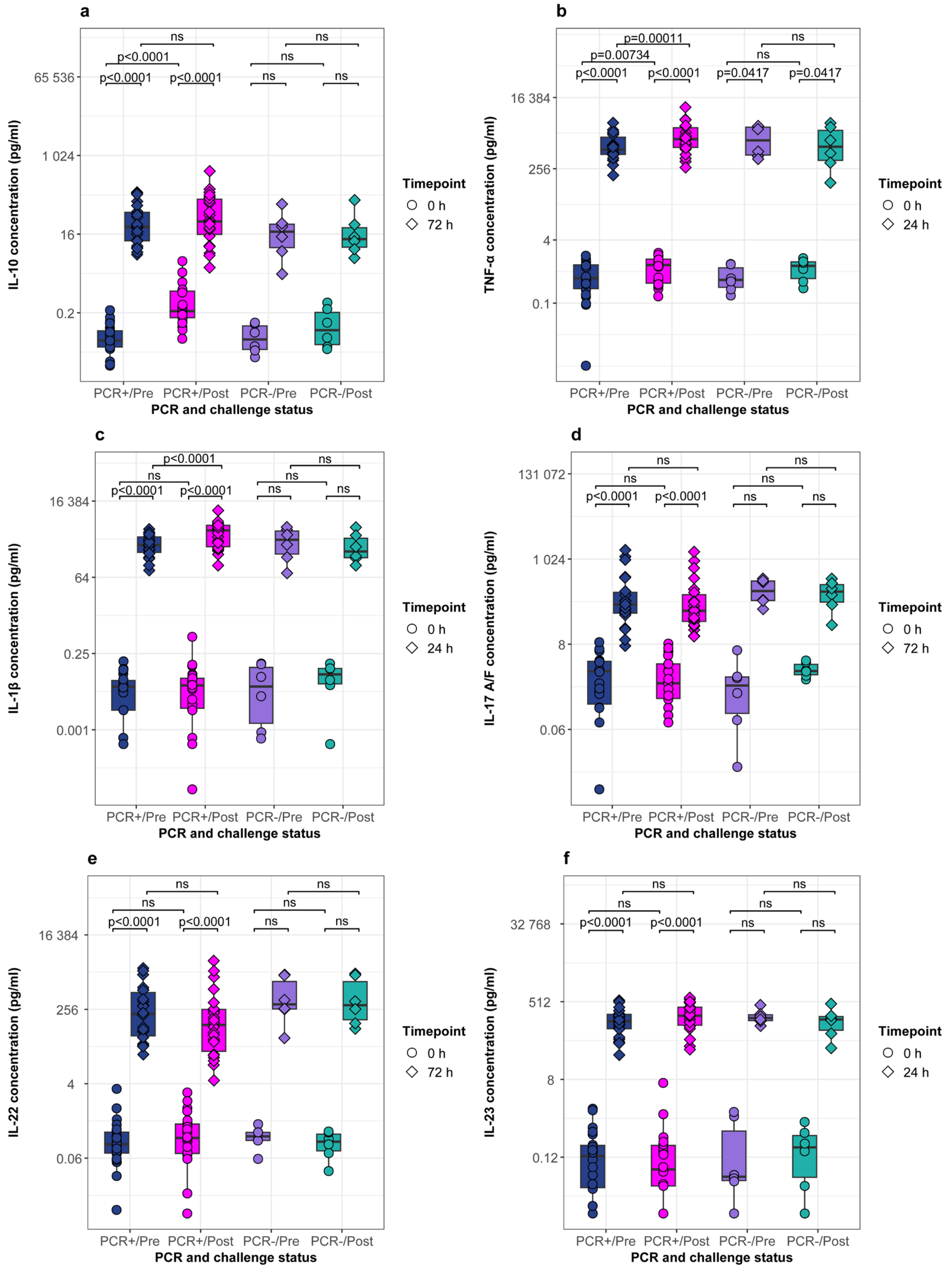

Supplementary Figure 4. Changing frequencies of cell subsets over time. Plots showing the frequency of **a** IFN-α+ intermediate monocytes, **b** IFN-α+ non-classical monocytes, **c** IFN-α+ NK cells, **d** TNF-α+ classical monocytes, **e** IL-10+ classical monocytes over time. For each cellular subtype, box and whiskers plots of frequency in BCG *lux*-uninfected blood at baseline (0h, circles) and BCG *lux*- uninfected and infected blood (triangles and squares respectively) at 6 h (c, d) and 24 h (a, b, e) are shown for 16 influenza PCR+ participants, before (blue) and after (pink) influenza infection, with the boxes representing the interquartile ranges (IQR), the horizontal lines showing the medians and whiskers denoting adjacent values within 1.5 IQR of the first and third quartiles. Paired comparisons of medians were made using two-tailed Wilcoxon rank sum between pre- versus post-influenza samples, and BCG *lux*-infected versus -uninfected samples. In all plots, false discovery rate (FDR)-corrected p values are shown; ns denotes adj. p >0.05. Source data are provided as a Source Data file.

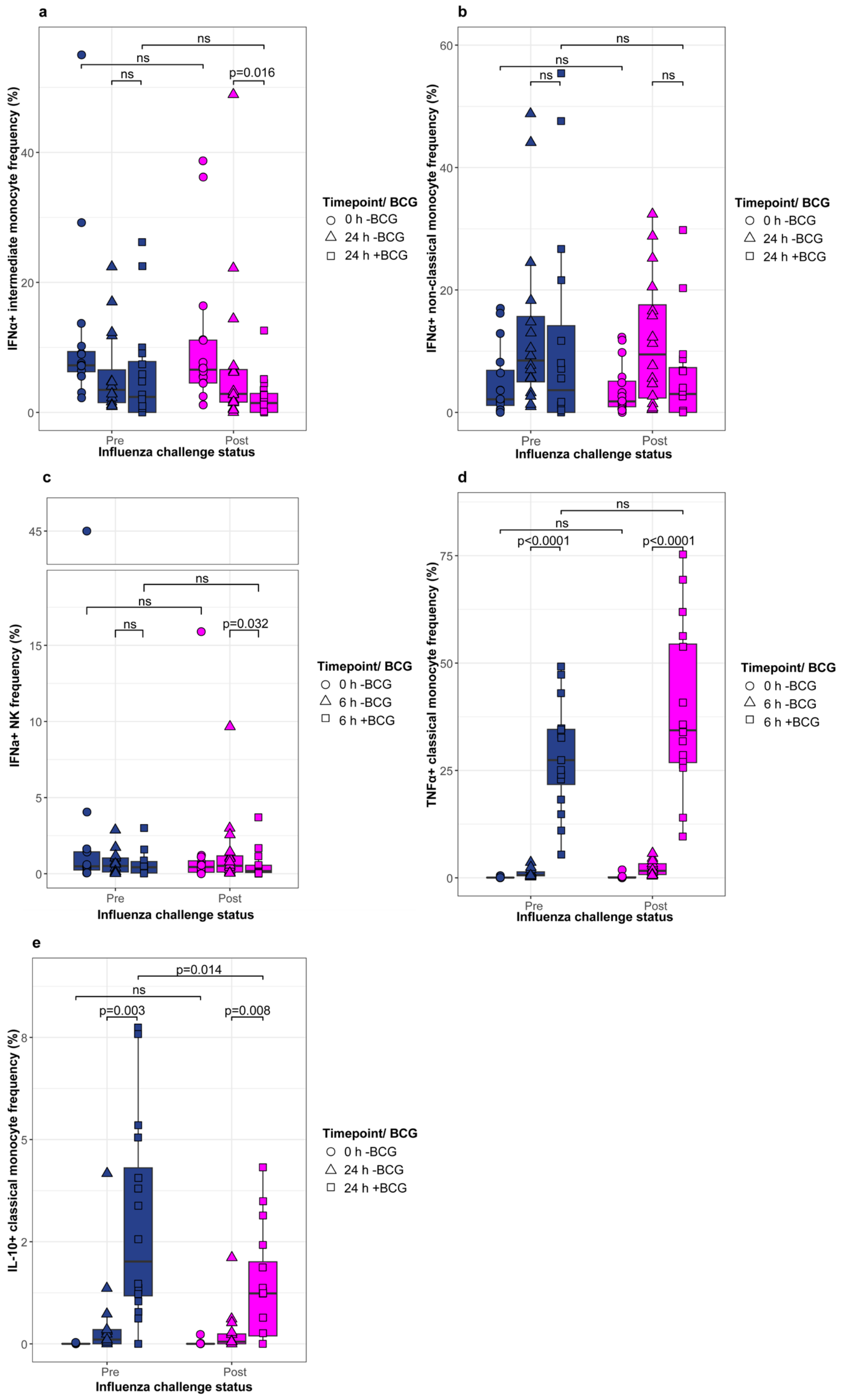

Supplementary Tables

Supplementary Table 1. Demographics of influenza challenge patients testing PCR-positive (+) and PCR-negative (-) for Influenza A (H3N2) virus. Sex and ethnicity were self-reported by participants. Two-tailed Wilcoxon and Fisher's exact tests of significance were employed for comparisons of medians. Source data are provided as a Source Data file.

	PCR+	PCR-	p
Total number of participants, n	24	6	
Age (median, IQR [years])	42.5 (33.6-51.4)	44.0 (37.4-50.6)	0.53
Female sex, n (%)	12 (50)	3 (50)	1
Male sex, n (%)	12 (50)	3 (50)	
Ethnicity, n (%) Asian	5 (21)	0	0.78
Black	2 (8)	0	
White	16 (67)	6 (100)	
Other	1 (4)	0	

Supplementary Table 2. Comparisons of baseline (pre-influenza) BCG *lux* $GR_{72\ h}$ and $\Delta GR_{72\ h}$ for demographics and viral load. Sex and ethnicity were self-reported by participants. Two-tailed Wilcoxon and Fisher's exact tests of significance were employed for comparisons of medians. Source data are provided as a Source Data file.

	Median/ Mean	Pearson's correlation coefficient	p value
$GR_{72\ h}$ (all, n=28)			
Sex (n)	F=1.35 (15), M=0.99 (13)		0.27
Ethnicity (n)	A=0.74 (5), B= 1.74 (2), O= 0.47 (1), W= 1.15 (20)		1
Age		0.04	0.84
$\Delta GR_{72\ h}$ (PCR+ only, n=22)			
Sex (n)	F=148 (12), M=147 (10)		0.93
Ethnicity (n)	A=172 (5), B= 130 (2), O= 169 (1), W= 153 (14)		0.89
Age		-0.24	0.29
Maximal viral load		0.1	0.65

Supplementary Table 3. Antibodies. Summary of antibodies used in experiments to quantify cytokines and cellular subsets. For cytokine quantification with U-plex kits, analytes were measured across two plates, with one plate measuring TNF-α, IFN-γ and IL-1β and the other plate measuring IL-10, IL-17A/F, IL-22, IL-23. All samples (U-plex and S-plex assays) were added neat, except for the 6 h, 24 h and 72 h samples on the TNF-α, IFN-γ and IL-1β plate, which were diluted 1:10 with the included kit assay diluent. For flow cytometry, all antibody dilutions were 1:100 except for PE anti -human IL-10 antibody which was 1:200.

Reagent	Source	Catalogue number
Quantification of cytokines		
U-PLEX Custom Biomarker Group 1 (human) Assay kit	Meso Scale Discovery	K15067L-1
Human IFN-γ antibody set	Meso Scale Discovery	B21TT-2
Human TNF-α antibody set	Meso Scale Discovery	B21UC-2
Human IL-1β antibody set	Meso Scale Discovery	B21TU-2
Human IL-10 antibody set	Meso Scale Discovery	B21TZ-2
Human IL-17A/F antibody set	Meso Scale Discovery	B21VY-2
Human IL-22 antibody set	Meso Scale Discovery	B21WI-2
Human IL-23 antibody set	Meso Scale Discovery	B21WG-2
S-PLEX Human IFN-α2a kit	Meso Scale Discovery	K151P3S
S-PLEX Human IFN-β kit	Meso Scale Discovery	K151ADRS
Flow cytometry antibodies		
Zombie NIR Fixable Viability kit	Biolegend	423106
FITC anti-human CD3 antibody, UCHT1 clone	Biolegend	300405
PerCP-Cy5.5 anti-human CD4 antibody, OKT4 clone	Biolegend	317427
PE anti -human IL-10 antibody, JES3-9D7 clone	Biolegend	501403
Alexa Fluor 647 anti-human IFN-γ antibody, 4SB3 clone	Biolegend	502516
Alexa Fluor 700 anti-human CD14 antibody, 63D3 clone	Biolegend	367113
Brilliant violet 605 anti-human CD16 antibody, 3G8 clone	Biolegend	302039
Brilliant violet 711 anti-human CD8a antibody, RPA-T8 clone	Biolegend	301043
Brilliant violet 421 anti-human TNFα antibody, Mab11 clone	Biolegend	502931
Brilliant Violet 785 anti-human HLA-DR antibody, L243 clone	Biolegend	307641
BV510 anti human CD56 antibody, NCAM16.2 clone	BD Biosciences	563041
PE-Vio 615 anti human IFN-α REAfinity antibody, REA1013 clone	Miltenyi Biotec	130-116-995

Supplementary References

1. Jiang H, Tsang L, Wang H, Liu C. IFI44L as a forward regulator enhancing host antituberculosis responses. *Journal of Immunology Research* **2021**, 5599408 (2021).

2. Singhania A*, et al.* A modular transcriptional signature identifies phenotypic heterogeneity of human tuberculosis infection. *Nature communications* **9**, 2308 (2018).

3. Yu J, Liu SL. Emerging Role of LY6E in Virus-Host Interactions. *Viruses* **11**, (2019).

4. Xu X*, et al.* IFN-stimulated gene LY6E in monocytes regulates the CD14/TLR4 pathway but inadequately restrains the hyperactivation of monocytes during chronic HIV-1 infection. *Journal of immunology (Baltimore, Md : 1950)* **193**, 4125-4136 (2014).

5. Hornung V, Hartmann R, Ablasser A, Hopfner K-P. OAS proteins and cGAS: unifying concepts in sensing and responding to cytosolic nucleic acids. *Nature Reviews Immunology* **14**, 521-528 (2014).

6. Berry MP*, et al.* An interferon-inducible neutrophil-driven blood transcriptional signature in human tuberculosis. *Nature* **466**, 973-977 (2010).

7. Maertzdorf J*, et al.* Human gene expression profiles of susceptibility and resistance in tuberculosis. *Genes & Immunity* **12**, 15-22 (2011).

8. Ottenhoff TH*, et al.* Genome-wide expression profiling identifies type 1 interferon response pathways in active tuberculosis. *PloS one* **7**, e45839 (2012).

9. Leisching G, Wiid I, Baker B. OAS1, 2, and 3: Significance During Active Tuberculosis? *The Journal of infectious diseases* **217**, 1517-1521 (2018).

10. Leisching G, Cole V, Ali AT, Baker B. OAS1, OAS2 and OAS3 restrict intracellular M. tb replication and enhance cytokine secretion. *International journal of infectious diseases : IJID : official publication of the International Society for Infectious Diseases* **80s**, S77-s84 (2019).

11. Helbig KJ, Beard MR. The Role of Viperin in the Innate Antiviral Response. *Journal of Molecular Biology* **426**, 1210-1219 (2014).

12. Helbig KJ*, et al.* The interferon stimulated gene viperin, restricts Shigella. flexneri in vitro. *Scientific Reports* **9**, 15598 (2019).

13. Manzanillo PS, Shiloh MU, Portnoy DA, Cox JS. Mycobacterium tuberculosis activates the DNA-dependent cytosolic surveillance pathway within macrophages. *Cell host & microbe* **11**, 469-480 (2012).

14. Abella V*, et al.* Leptin in the interplay of inflammation, metabolism and immune system disorders. *Nature Reviews Rheumatology* **13**, 100-109 (2017).

15. Naylor C, Petri WA, Jr. Leptin Regulation of Immune Responses. *Trends in molecular medicine* **22**, 88-98 (2016).

16. Wieland CW*, et al.* Pulmonary Mycobacterium tuberculosis infection in leptin-deficient ob/ob mice. *International Immunology* **17**, 1399-1408 (2005).

17. Chen Y, Cao S, Sun Y, Li C. Gene expression profiling of the TRIM protein family reveals potential biomarkers for indicating tuberculosis status. *Microbial pathogenesis* **114**, 385-392 (2018).

18. van Tol S*, et al.* VAMP8 Contributes to the TRIM6-Mediated Type I Interferon Antiviral Response during West Nile Virus Infection. *Journal of virology* **94**, (2020)

19. Villamayor L*, et al.* Interferon alpha inducible protein 6 is a negative regulator of innate immune responses by modulating RIG-I activation. *Frontiers in immunology* **14**, 1105309 (2023).

20. Mariotti B*, et al.* The Long Non-coding RNA NRIR Drives IFN-Response in Monocytes: Implication for Systemic Sclerosis. *Frontiers in immunology* **10**, 100 (2019).

21. Ren T*, et al.* ID1 inhibits foot-and-mouth disease virus replication via targeting of interferon pathways. *The FEBS Journal* **288**, 4364-4381 (2021).

22. Roy S*, et al.* Transcriptional landscape of Mycobacterium tuberculosis infection in macrophages. *Scientific Reports* **8**, 6758 (2018).

23. Verhelst J, Parthoens E, Schepens B, Fiers W, Saelens X. Interferon-Inducible Protein Mx1 Inhibits Influenza Virus by Interfering with Functional Viral Ribonucleoprotein Complex Assembly. *Journal of virology* **86**, 13445-13455 (2012).

24. Kim Y-H, Lee J-R, Hahn M-J. Regulation of inflammatory gene expression in macrophages by epithelial-stromal interaction 1 (Epsti1). *Biochemical and Biophysical Research Communications* **496**, 778-783 (2018).

25. Wu S, Wang Y, Chen G, Zhang M, Wang M, He JQ. 2'-5'-Oligoadenylate synthetase 1 polymorphisms are associated with tuberculosis: a case-control study. *BMC pulmonary medicine* **18**, 180 (2018).

26. Campbell JA, Lenschow DJ. Emerging roles for immunomodulatory functions of free ISG15. *Journal of interferon & cytokine research : the official journal of the International Society for Interferon and Cytokine Research* **33**, 728-738 (2013).

27. Bogunovic D*, et al.* Mycobacterial disease and impaired IFN-γ immunity in humans with inherited ISG15 deficiency. *Science* **337**, 1684-1688 (2012).

28. Pohl C, Dikic I. Fighting mycobacteria through ISGylation. *EMBO reports* **13**, 872-873 (2012).

29. Xu D*, et al.* PLSCR1 is a cell-autonomous defence factor against SARS-CoV-2 infection. *Nature* **619**, 819-827 (2023).

30. Yang AX, Norbrun C, Sorkhdini P, Zhou Y. Phospholipid scramblase 1: a frontline defense against viral infections. *Frontiers in cellular and infection microbiology* **15**, (2025).

31. Arias CF*, et al.* p21CIP1/WAF1 Controls Proliferation of Activated/Memory T Cells and Affects Homeostasis and Memory T Cell Responses1. *The Journal of Immunology* **178**, 2296-2306 (2007).

32. Lloberas J, Celada A. p21waf1/CIP1, a CDK inhibitor and a negative feedback system that controls macrophage activation. *European journal of immunology* **39**, 691-694 (2009).

33. Scatizzi JC*, et al.* The CDK domain of p21 is a suppressor of IL-1β-mediated inflammation in activated macrophages. *European journal of immunology* **39**, 820-825 (2009).

34. Lv X*, et al.* RTP4 restricts influenza A virus infection by targeting the viral NS1 protein. *Virology* **603**, 110397 (2025).

35. He X*, et al.* RTP4 inhibits IFN-I response and enhances experimental cerebral malaria and neuropathology. *Proceedings of the National Academy of Sciences of the United States of America* **117**, 19465-19474 (2020).

36. Zanoni I, Granucci F. Role of CD14 in host protection against infections and in metabolism regulation. *Frontiers in cellular and infection microbiology* **3**, 32 (2013).

37. Burel JG*, et al.* Profiling the myeloid compartment of PBMC in active tuberculosis reveals substantial changes in CD14+ cells and upregulation of CD16 in pro-inflammatory dendritic cells. *The Journal of Immunology* **208**, 161.102-161.102 (2022).

38. Oshiumi H*, et al.* DDX60 Is Involved in RIG-I-Dependent and Independent Antiviral Responses, and Its Function Is Attenuated by Virus-Induced EGFR Activation. *Cell reports* **11**, 1193-1207 (2015).

39. Luo L*, et al.* SCIMP is a transmembrane non-TIR TLR adaptor that promotes proinflammatory cytokine production from macrophages. *Nature communications* **8**, 14133 (2017).

40. Liu L*, et al.* The transmembrane adapter SCIMP recruits tyrosine kinase Syk to phosphorylate Toll-like receptors to mediate selective inflammatory outputs. *Journal of Biological Chemistry* **298**, 101857 (2022).

41. Yang H, Winkler W, Wu X. Interferon Inducer IFI35 Regulates RIG-I-Mediated Innate Antiviral Response through Mutual Antagonism with Influenza Virus Protein NS1. *Journal of virology* **95**, 10.1128/jvi.00283-00221 (2021).

42. Xiahou Z*, et al.* NMI and IFP35 serve as proinflammatory DAMPs during cellular infection and injury. *Nature communications* **8**, 950 (2017).

43. Honda K*, et al.* IRF-7 is the master regulator of type-I interferon-dependent immune responses. *Nature* **434**, 772-777 (2005).

44. Jefferies CA. Regulating IRFs in IFN Driven Disease. *Frontiers in immunology* **10**, 325 (2019).

45. Zhang Q*, et al.* Inborn errors of type I IFN immunity in patients with life-threatening COVID-19. *Science* **370**, (2020).

46. Ciancanelli MJ*, et al.* Infectious disease. Life-threatening influenza and impaired interferon amplification in human IRF7 deficiency. *Science* **348**, 448-453 (2015).

47. Zhang G*, et al.* An SNP selection strategy identified IL-22 associating with susceptibility to tuberculosis in Chinese. *Sci Rep* **1**, 20 (2011).

48. Cheng Y, Schorey JS. Mycobacterium tuberculosis-induced IFN-β production requires cytosolic DNA and RNA sensing pathways. *The Journal of experimental medicine* **215**, 2919-2935 (2018).

49. Leisching G, Pietersen RD, van Heerden C, van Helden P, Wiid I, Baker B. RNAseq reveals hypervirulence-specific host responses to M. tuberculosis infection. *Virulence* **8**, 848-858 (2017).

50. Zhang Z*, et al.* MicroRNA-31 mediated by interferon regulatory factor 7 signaling facilitates control of Mycobacterium tuberculosis infection. *International journal of medical microbiology : IJMM* **312**, 151569 (2022).

51. Cubillos-Angulo JM*, et al.* Polymorphisms in interferon pathway genes and risk of Mycobacterium tuberculosis infection in contacts of tuberculosis cases in Brazil. *International journal of infectious diseases : IJID : official publication of the International Society for Infectious Diseases* **92**, 21-28 (2020).

52. Velie BD*, et al.* Using an Inbred Horse Breed in a High Density Genome-Wide Scan for Genetic Risk Factors of Insect Bite Hypersensitivity (IBH). *PloS one* **11**, e0152966 (2016).

53. Xie F, Dong H, Zhang H. Regulatory Functions of Protein Tyrosine Phosphatase Receptor Type O in Immune Cells. *Frontiers in immunology* **12**, 783370 (2021).

54. Du X*, et al.* Noncanonical Role of FBXO6 in Regulating Antiviral Immunity. *The Journal of Immunology* **203**, 1012-1020 (2019).

55. Martínez-Ramos S*, et al.* Semaphorin3B promotes an anti-inflammatory and pro-resolving phenotype in macrophages from rheumatoid arthritis patients in a MerTK-dependent manner. *Frontiers in immunology* **14**, 1268144 (2024).

56. McCormack RM*, et al.* Perforin-2 is essential for intracellular defense of parenchymal cells and phagocytes against pathogenic bacteria. *eLife* **4**, e06508 (2015).

57. McCormack RM*, et al.* MPEG1/perforin-2 mutations in human pulmonary nontuberculous mycobacterial infections. *JCI insight* **2**, (2017).

58. Jeong G*, et al.* A Kelch domain-containing KLHDC7B and a long non-coding RNA ST8SIA6-AS1 act oppositely on breast cancer cell proliferation via the interferon signaling pathway. *Scientific Reports* **8**, 12922 (2018).

59. Yi E, Oh J, Kang HR, Song MJ, Park SH. BST2 inhibits infection of influenza A virus by promoting apoptosis of infected cells. *Biochem Biophys Res Commun* **509**, 414-420 (2019).

60. Poladian N*, et al.* Role of NF-κB during Mycobacterium tuberculosis Infection. *International journal of molecular sciences* **24**, (2023).

61. Tiwari R, de la Torre JC, McGavern DB, Nayak D. Beyond Tethering the Viral Particles: Immunomodulatory Functions of Tetherin (BST-2). *DNA and cell biology* **38**, 1170-1177 (2019).

62. Stafford CA*, et al.* Phosphorylation of muramyl peptides by NAGK is required for NOD2 activation. *Nature* **609**, 590-596 (2022).

63. von Bernuth H*, et al.* Pyogenic Bacterial Infections in Humans with MyD88 Deficiency. *Science* **321**, 691-696 (2008).

64. O'Garra A, Redford PS, McNab FW, Bloom CI, Wilkinson RJ, Berry MP. The immune response in tuberculosis. *Annual review of immunology* **31**, 475-527 (2013).

65. Jiang Z*, et al.* IFI16 directly senses viral RNA and enhances RIG-I transcription and activation to restrict influenza virus infection. *Nature Microbiology* **6**, 932-945 (2021).

66. Unterholzner L*, et al.* IFI16 is an innate immune sensor for intracellular DNA. *Nature Immunology* **11**, 997-1004 (2010).

67. Zschiedrich I*, et al.* Coactivator function of RIP140 for NFκB/RelA-dependent cytokine gene expression. *Blood* **112**, 264-276 (2008).

68. Cao L*, et al.* The RNA-Splicing Ligase RTCB Promotes Influenza A Virus Replication by Suppressing Innate Immunity via Interaction with RNA Helicase DDX1. *Journal of immunology (Baltimore, Md : 1950)* **211**, 1020-1031 (2023).

69. Mandatori S*, et al.* PRKAG2.2 is essential for FoxA1(+) regulatory T cell differentiation and metabolic rewiring distinct from FoxP3(+) regulatory T cells. *Sci Adv* **9**, eadj8442 (2023).

70. Jiao S*, et al.* The kinase MST4 limits inflammatory responses through direct phosphorylation of the adaptor TRAF6. *Nature Immunology* **16**, 246-257 (2015).
