# Supplementary material for: Influenza coinfection inhibits control of mycobacterial infection in a human challenge model
Source: Nat Commun. 2026 Jun 11;17:4884. doi: 10.1038/s41467-026-72363-2 (PMC13261052; doi:10.1038/s41467-026-72363-2)
Supplement: Supplementary file 2 — Description of Additional Supplementary Files [file 41467_2026_72363_MOESM2_ESM.pdf]

Title: Supplementary Data 1

Description: Genes significantly differentially expressed for the BCG: Influenza interaction and their associations with anti-mycobacterial immune responses. Differential expression analysis was performed using the DESeq2 package. Statistical significance was determined using a two-sided Wald test to assess whether the log2-fold change for a given comparison was different from zero. The resulting p-values were adjusted for multiple comparisons using the Benjamini Hochberg (BH) method. Significance threshold was set at adjusted $p \leq 0.05$.

Title: Supplementary Data 2

Description: Significantly differentially expressed genes identified in DESeq2 for the variable BCG. Differential expression analysis was performed using the DESeq2 package. Statistical significance was determined using a two-sided Wald test to assess whether the log2-fold change for a given comparison was different from zero. The resulting p-values were adjusted for multiple comparisons using the Benjamini-Hochberg (BH) method. Significance threshold was set at adjusted $p \leq 0.05$.

Title: Supplementary Data 3

Description: Significantly differentially expressed genes identified in DESeq2 for the variable Influenza. Differential expression analysis was performed using the DESeq2 package. Statistical significance was determined using a two-sided Wald test to assess whether the log2-fold change for a given comparison was different from zero. The resulting p-values were adjusted for multiple comparisons using the Benjamini-Hochberg (BH) method. Significance threshold was set at adjusted $p \leq 0.05$.

Title: Supplementary Data 4

Description: Significant pathways identified using IPA for BCG: Influenza. Significantly differentially expressed genes identified for BCG: Influenza were subjected to pathway analysis using Ingenuity Pathway Analysis (IPA). Gene filters for IPA analysis: adj $p \leq 0.05$. P-values from the IPA pathway analysis were adjusted for multiple comparisons using the Benjamini-Hochberg (BH) method, with a significance threshold set at adjusted $p \leq 0.05$. Z-scores are independent from p-values and indicate the predicted direction and magnitude of change of the pathway: higher absolute z-scores indicate a larger degree of change; positive and negative z-scores indicate activation and repression respectively. Where IPA cannot predict the directionality, no Z score is given. The ratio is the number of molecules in a given pathway that meet threshold criteria, divided by the total number of molecules that make up that pathway and are in the reference set.

Title: Supplementary Data 5

Description: Significant pathways identified using IPA for BCG. Significantly differentially expressed genes identified for BCG were subjected to pathway analysis using Ingenuity Pathway Analysis (IPA). Gene filters for IPA analysis: adj p ≤0.0001 and absolute LFC >1. P-values from the IPA pathway analysis were adjusted for multiple comparisons using the Benjamini-Hochberg (BH) method, with a significance threshold set at adjusted p ≤ 0.05. Z-scores are independent from p-values and indicate the predicted direction and magnitude of change of the pathway: higher absolute z-scores indicate a larger degree of change; positive and negative z-scores indicate activation and repression respectively. Where IPA cannot predict the directionality, no Z score is given. The ratio is the number of molecules in a given pathway that meet threshold criteria, divided by the total number of molecules that make up that pathway and are in the reference set.

Title: Supplementary Data 6

Description: Significant pathways identified using IPA for Influenza. Significantly differentially expressed genes identified for Influenza were subjected to pathway analysis using Ingenuity Pathway Analysis (IPA). Gene filters for IPA analysis: adj p ≤0.0001 and absolute LFC >0.5. P values from the IPA pathway analysis were adjusted for multiple comparisons using the Benjamini Hochberg (BH) method, with a significance threshold set at adjusted p ≤ 0.05. Z-scores are independent from p-values and indicate the predicted direction and magnitude of change of the pathway: higher absolute z-scores indicate a larger degree of change; positive and negative z-scores indicate activation and repression respectively. Where IPA cannot predict the directionality, no Z score is given. The ratio is the number of molecules in a given pathway that meet threshold criteria, divided by the total number of molecules that make up that pathway and are in the reference set.

Title: Supplementary Data 7

Description: Significantly differentially expressed genes identified in DESeq2 with the cell adjusted model for the BCG: Influenza interaction. Differential expression analysis was performed using the DESeq2 package. Statistical significance was determined using a two-sided Wald test to assess whether the log2-fold change for a given comparison was different from zero. The resulting p values were adjusted for multiple comparisons using the Benjamini-Hochberg (BH) method. Significance threshold was set at adjusted p ≤0.05.

Title: Supplementary Data 8

Description: Significantly differentially expressed genes identified in DESeq2 with the cell adjusted model for the variable BCG. Differential expression analysis was performed using the DESeq2 package. Statistical significance was determined using a two-sided Wald test to assess whether the log2-fold change for a given comparison was

different from zero. The resulting p-values were adjusted for multiple comparisons using the Benjamini-Hochberg (BH) method. Significance threshold was set at adjusted p ≤ 0.05.

Title: Supplementary Data 9

Description: Significantly differentially expressed genes identified in DESeq2 with the cell adjusted model for the variable Influenza. Differential expression analysis was performed using the DESeq2 package. Statistical significance was determined using a two-sided Wald test to assess whether the log2-fold change for a given comparison was different from zero. The resulting p-values were adjusted for multiple comparisons using the Benjamini-Hochberg (BH) method. Significance threshold was set at adjusted p ≤ 0.05.

Title: Supplementary Data 10

Description: Significant pathways identified using IPA for BCG: Influenza (cell-adjusted model). Significantly differentially expressed genes identified for BCG: Influenza were subjected to pathway analysis using Ingenuity Pathway Analysis (IPA). Gene filters for IPA analysis: adj p ≤ 0.05. P values from the IPA pathway analysis were adjusted for multiple comparisons using the Benjamini Hochberg (BH) method, with a significance threshold set at adjusted $p \leq 0.05$. Z-scores are independent from p-values and indicate the predicted direction and magnitude of change of the pathway: higher absolute z-scores indicate a larger degree of change; positive and negative z-scores indicate activation and repression respectively. Where IPA cannot predict the directionality, no Z score is given. The ratio is the number of molecules in a given pathway that meet threshold criteria, divided by the total number of molecules that make up that pathway and are in the reference set.

Title: Supplementary Data 11

Description: Significant pathways identified using IPA for BCG (cell-adjusted model). Significantly differentially expressed genes identified for BCG were subjected to pathway analysis using Ingenuity Pathway Analysis (IPA). Gene filters for IPA analysis: adj p ≤ 0.01 and absolute LFC >0.5. P values from the IPA pathway analysis were adjusted for multiple comparisons using the Benjamini Hochberg (BH) method, with a significance threshold set at adjusted $p \leq 0.05$. Z-scores are independent from p-values and indicate the predicted direction and magnitude of change of the pathway: higher absolute z-scores indicate a larger degree of change; positive and negative z-scores indicate activation and repression respectively. Where IPA cannot predict the directionality, no Z score is given. The ratio is the number of molecules in a given pathway that meet threshold criteria, divided by the total number of molecules that make up that pathway and are in the reference set.

Title: Supplementary Data 12

Description: Significant pathways identified using IPA for Influenza (cell-adjusted model). Significantly differentially expressed genes identified for Influenza were subjected to pathway analysis using Ingenuity Pathway Analysis (IPA). Gene filters for IPA analysis: adj p ≤0.01 and absolute LFC >0.5. P-values from the IPA pathway analysis were adjusted for multiple comparisons using the Benjamini-Hochberg (BH) method, with a significance threshold set at adjusted p ≤ 0.05. Z-scores are independent from p-values and indicate the predicted direction and magnitude of change of the pathway: higher absolute z-scores indicate a larger degree of change; positive and negative z-scores indicate activation and repression respectively. Where IPA cannot predict the directionality, no Z score is given. The ratio is the number of molecules in a given pathway that meet threshold criteria, divided by the total number of molecules that make up that pathway and are in the reference set.

Title: Supplementary Data 13

Description: Significantly differentially expressed genes identified by maSigPro. Analysis of time-course data was performed using the maSigPro package, which uses a two-step polynomial regression approach. Significance of temporal changes and differences between groups was assessed using an F-test. P-values from this test were adjusted for multiple comparisons using the Benjamini Hochberg (BH) method. Significance threshold was set at adj p ≤ 0.05, R2>0.6. Column "Gene cluster" denotes membership of the 9 gene clusters where applicable.

Title: Supplementary Data 14

Description: Significant pathways identified using IPA for Cluster 1. Significantly differentially expressed genes identified for Cluster 1 were subjected to pathway analysis using Ingenuity Pathway Analysis (IPA). Gene filters for IPA analysis: adj p ≤0.05. P-values from the IPA pathway analysis were adjusted for multiple comparisons using the Benjamini-Hochberg (BH) method, with a significance threshold set at adj p ≤ 0.05. The ratio is the number of molecules in a given pathway that meet threshold criteria, divided by the total number of molecules that make up that pathway and are in the reference set.

Title: Supplementary Data 15

Description: Significant pathways identified using IPA for Cluster 2. Significantly differentially expressed genes identified for Cluster 2 were subjected to pathway analysis using Ingenuity Pathway Analysis (IPA). Gene filters for IPA analysis: adj p ≤0.05. P-values from the IPA pathway analysis were adjusted for multiple comparisons using the Benjamini-Hochberg (BH) method, with a significance threshold set at adj p ≤ 0.05. The ratio is the number of molecules in a given pathway that meet threshold

criteria, divided by the total number of molecules that make up that pathway and are in the reference set.

Title: Supplementary Data 16

Description: Significant pathways identified using IPA for Cluster 3. Significantly differentially expressed genes identified for Cluster 3 were subjected to pathway analysis using Ingenuity Pathway Analysis (IPA). Gene filters for IPA analysis: adj p ≤ 0.05. P-values from the IPA pathway analysis were adjusted for multiple comparisons using the Benjamini-Hochberg (BH) method, with a significance threshold set at adj p $\leq$ 0.05. The ratio is the number of molecules in a given pathway that meet threshold criteria, divided by the total number of molecules that make up that pathway and are in the reference set.

Title: Supplementary Data 17

Description: Significant pathways identified using IPA for Cluster 4. Significantly differentially expressed genes identified for Cluster 4 were subjected to pathway analysis using Ingenuity Pathway Analysis (IPA). Gene filters for IPA analysis: adj p ≤ 0.05. P-values from the IPA pathway analysis were adjusted for multiple comparisons using the Benjamini-Hochberg (BH) method, with a significance threshold set at adj p $\leq$ 0.05. The ratio is the number of molecules in a given pathway that meet threshold criteria, divided by the total number of molecules that make up that pathway and are in the reference set.

Title: Supplementary Data 18

Description: Significant pathways identified using IPA for Cluster 5. Significantly differentially expressed genes identified for Cluster 5 were subjected to pathway analysis using Ingenuity Pathway Analysis (IPA). Gene filters for IPA analysis: adj p ≤ 0.05. P-values from the IPA pathway analysis were adjusted for multiple comparisons using the Benjamini-Hochberg (BH) method, with a significance threshold set at adj p $\leq$ 0.05. The ratio is the number of molecules in a given pathway that meet threshold criteria, divided by the total number of molecules that make up that pathway and are in the reference set.

Title: Supplementary Data 19

Description: Significant pathways identified using IPA for Cluster 6. Significantly differentially expressed genes identified for Cluster 6 were subjected to pathway analysis using Ingenuity Pathway Analysis (IPA). Gene filters for IPA analysis: adj p ≤ 0.05. P-values from the IPA pathway analysis were adjusted for multiple comparisons using the Benjamini-Hochberg (BH) method, with a significance threshold set at adj p $\leq$ 0.05. The ratio is the number of molecules in a given pathway that meet threshold

criteria, divided by the total number of molecules that make up that pathway and are in the reference set.

Title: Supplementary Data 20

Description: Significant pathways identified using IPA for Cluster 8. Significantly differentially expressed genes identified for Cluster 8 were subjected to pathway analysis using Ingenuity Pathway Analysis (IPA). Gene filters for IPA analysis: adj p ≤0.05. P-values from the IPA pathway analysis were adjusted for multiple comparisons using the Benjamini-Hochberg (BH) method, with a significance threshold set at adj p ≤ 0.05. The ratio is the number of molecules in a given pathway that meet threshold criteria, divided by the total number of molecules that make up that pathway and are in the reference set.
