# Supplementary material for: Influenza coinfection inhibits control of mycobacterial infection in a human challenge model
Source: Nat Commun. 2026 Jun 11;17:4884. doi: 10.1038/s41467-026-72363-2 (PMC13261052; doi:10.1038/s41467-026-72363-2)
Supplement: Supplementary file 23 — Reporting summary [file 41467_2026_72363_MOESM23_ESM.pdf]

nature portfolio

Corresponding author(s): Claire Broderick

Last updated by author(s): May 30, 2025

Reporting Summary

Nature Portfolio wishes to improve the reproducibility of the work that we publish. This form provides structure for consistency and transparency in reporting. For further information on Nature Portfolio policies, see our Editorial Policies and the Editorial Policy Checklist.

Statistics

For all statistical analyses, confirm that the following items are present in the figure legend, table legend, main text, or Methods section.

n/a	Confirmed	
☐	☒	The exact sample size (*n*) for each experimental group/condition, given as a discrete number and unit of measurement
☐	☒	A statement on whether measurements were taken from distinct samples or whether the same sample was measured repeatedly
☐	☒	The statistical test(s) used AND whether they are one- or two-sided<br>*Only common tests should be described solely by name; describe more complex techniques in the Methods section.*
☐	☒	A description of all covariates tested
☐	☒	A description of any assumptions or corrections, such as tests of normality and adjustment for multiple comparisons
☐	☒	A full description of the statistical parameters including central tendency (e.g. means) or other basic estimates (e.g. regression coefficient) AND variation (e.g. standard deviation) or associated estimates of uncertainty (e.g. confidence intervals)
☐	☒	For null hypothesis testing, the test statistic (e.g. *F*, *t*, *r*) with confidence intervals, effect sizes, degrees of freedom and *P* value noted<br>*Give P values as exact values whenever suitable.*
☒	☐	For Bayesian analysis, information on the choice of priors and Markov chain Monte Carlo settings
☒	☐	For hierarchical and complex designs, identification of the appropriate level for tests and full reporting of outcomes
☒	☐	Estimates of effect sizes (e.g. Cohen's *d*, Pearson's *r*), indicating how they were calculated

Our web collection on statistics for biologists contains articles on many of the points above.

Software and code

Policy information about availability of computer code

Data collection	Sample acquisition for flow cytometry utillised FACSDiva software (version 9.2, BD Biosciences).<br>MSD Methodical Mind Analysis software (MSD) was used to acquire cytokine data.
Data analysis	BCG lux GR72 h and ΔGR72 h were plotted and analysed in GraphPad Prism (version 9 for Windows, GraphPad Software, www.graphpad.com).<br><br>MSD discovery workbench software (version 4.0) was used to analyse cytokine data.<br><br>FlowJo (version 10.10.0, Ashland, OR, USA) was used to analyse flow cytometry data<br><br>Code for the gene expression analyses is available on Github: https://github.com/cmbroderick/bcg_influenza.<br>Following annotation with the R package biomaRt, differential expression analyses were performed using the DESeq2 and maSigPro R packages. Wilcoxon rank sum and t tests were performed using rstatix. Graphs were plotted using the ggplot2. The heat map was plotted using the pheatmap package in R.<br><br>A complete list of the software/ R packages used in the analyses are listed below:<br>R (version 4.0.4).<br>biomaRt (version 2.46.3, accessed database Sept 2022). https://bioconductor.org/packages/release/bioc/html/biomaRt.html.<br>DESeq2 (version 1.30.1). https://bioconductor.org/packages/release/bioc/html/DESeq2.html.<br>maSigPro (version 1.78.0). https://www.bioconductor.org/packages/release/bioc/html/maSigPro.html.

rstatix (version 0.7.2). https://cran.r-project.org/web/packages/rstatix/index.html.
ggplot2 (version 3.5.1) https://cran.r-project.org/web/packages/ggplot2/index.html.
ggsignif (version 0.6.4) https://cran.r-project.org/web/packages/ggsignif/.
pheatmap (version 1.0.12) https://cran.r-project.org/web/packages/pheatmap/.
ggbreak (version 0.1.4) https://cran.r-project.org/web/packages/ggbreak/index.html.
gridExtra (version 2.3) (Auguie, B. & Antonov, A. gridExtra: Miscellaneous Functions for "Grid" Graphics, 2017).
cowplot (version 1.1.3) (Wilke, C.O. cowplot – Streamlined plot theme and plot annotations for ggplot2, 2016).
dplyr (version 1.1.4) Wickham H, F.R., Henry L, Müller K, Vaughan D. dplyr: A Grammar of Data Manipulation. (2023).
tidyr (version 1.3.1) Wickham H, V.D., Girlich M tidyr: Tidy Messy Data. (2023).
scales (version 1.4.0). Wickham H, P.T., Seidel D. scales: Scale Functions for Visualization. (2023).

Estimation of immune cell-type fractions from the bulk host transcriptome data was performed using CIBERSORTx (https://cibersortx.stanford.edu/).
Protein-protein interaction (PPI) networks were constructed using the Search Tool for the Retrieval of Interacting Genes (STRING) online database (https://string-db.org/) and the network model visualised using Cytoscape (V3.9.1).

For manuscripts utilizing custom algorithms or software that are central to the research but not yet described in published literature, software must be made available to editors and reviewers. We strongly encourage code deposition in a community repository (e.g. GitHub). See the Nature Portfolio guidelines for submitting code & software for further information.

Data

Policy information about availability of data

All manuscripts must include a data availability statement. This statement should provide the following information, where applicable:

- Accession codes, unique identifiers, or web links for publicly available datasets
- A description of any restrictions on data availability
- For clinical datasets or third party data, please ensure that the statement adheres to our policy

Processed RNAseq data are publicly available at ArrayExpress (accession number E-MTAB-15137).
Raw sequencing data are available under managed access through the European Genome-Phenome Archive (https://ega-archive.org), under accession number EGAS50000001677. Data will be shared with investigators whose proposed use is within the scope of participant consent, on request to the corresponding author, subject to a data access agreement.
Cytokine and flow cytometry data are provided in a Source data file.

Research involving human participants, their data, or biological material

Policy information about studies with human participants or human data. See also policy information about sex, gender (identity/presentation), and sexual orientation and race, ethnicity and racism.

Reporting on sex and gender	Male and female participants were included in the study. Sex was determined by self-reporting by participants. The study comprised of 30 participants, 15 female, 15 male. The proportions of males and females who developed influenza infection (i.e. PCR+) is reported (Supplementary table 1). There were no significant differences in mycobacterial growth restriction between males and females (Supplementary table 2). These data are included in the Source Data file.
Reporting on race, ethnicity, or other socially relevant groupings	Ethnicity was determined by self-reporting by participants, with 73% White and 27% Asian, Black or Other. Reporting of sex, age and ethnicity are provided for influenza PCR+ and PCR- participants (Supplementary table 1). Mycobacterial growth restriction did not differ significantly with sex, age or ethnicity (Supplementary table 2). Data are including in the Source Data file.
Population characteristics	Summary level data on participant characteristics is available in Supplementary table 1.
Recruitment	Healthy persons aged 18 to 55 years, able to give informed consent, were eligible for the study. Subjects were recruited via advertisement. Exclusion criteria included chronic respiratory disease, recent upper respiratory infection, immune deficiency, pregnancy, and close domestic contact with high-risk populations. These details are described in Temple et al, JID 2023; 227 (7):864–872, https://doi.org/10.1093/infdis/jiac262.<br>Written informed consent was obtained for all study participants.<br>Participants were compensated for the time and inconvenience of taking part in the study including study visits and quarantine. Full details of compensation are available in the study protocols and were approved by the Research Ethics Committee.
Ethics oversight	Regulatory approvals were provided by the UK Health Research Authority approval and the Brighton & Sussex Research Ethics Committee, London (reference 19/LO/0208) and London- Fulham Research Ethics Committee (references 11/LO/1826 and 19/LO/1441). This information is provided in the manuscript.

Note that full information on the approval of the study protocol must also be provided in the manuscript.

Field-specific reporting

For a reference copy of the document with all sections, see nature.com/documents/nr-reporting-summary-flat.pdf

Life sciences study design

All studies must disclose on these points even when the disclosure is negative.

Sample size	The present study did not form the basis of sample size calculations. The sample size for this study was determined by the availability of whole blood samples from influenza challenge participants, for use in mycobacterial growth inhibition assays and related transcriptional profiling and immune assays.
Data exclusions	Two participants were excluded from all analyses due to incomplete BCG lux growth data. The remaining 28 participants are included in the manuscript and the Source Data files.<br>All 28 participants (22 Influenza PCR+, 6 PCR-) were included in the mycobacterial growth inhibition and cytokine analyses.<br>All influenza PCR+ participants with complete sample sets (pre- and post-influenza challenge, 0 h, 6 h, 24 h time points) were included in the RNA-seq (n=15) and flow cytometry (n=16) experiments, to enable paired analyses.
Replication	Standardized sampling, storage and laboratory techniques were used for all patient samples.<br>All analyses included data from multiple participants, as indicated in the Results section.<br><br>For the mycobacterial growth inhibition assay: per participant per condition (per/post-influenza inoculation), triplicate blood aliquots were removed from the incubator at random per time point, and BCG lux luminescence was measured in duplicate for each aliquot, to give 6 luminescence readings from which the median was calculated; the medians were used for the growth ratio calculations.<br><br>For transcriptional profiling: Batch effects were minimised by the random allocation of all samples for RNA extractions, and a second random allocation of extracted samples for plating for sequencing. All three plates were sequenced together at the same sequencing centre. Principle component analyses (PCA) were performed to detect any batch effects from extraction or sequencing.<br><br>For MSD cytokine analyses: measurements were performed in duplicate and the mean of the two values was used. Participants were randomised to plates, with all samples from an individual participant included on the same plate due to the planned paired analyses. The location of each sample on the plates was randomly allocated. Positive and negative controls were included on the plates.<br><br>For flow cytometry: all samples from an individual participant were in the same run due to the planned paired analyses, with randomisation of run allocation and the order of samples.<br><br>The results from the transcriptional profiling, cytokine and flow cytometry analyses are consistent and support the BCG lux growth data.
Randomization	There was no a priori allocation of participants to separate groups. Between-group analyses was based on (i) presence or absence of replicative influenza infection; (ii) increase or decrease in mycobacterial growth restriction following influenza infection.
Blinding	Mycobacterial growth inhibition assays: Investigators were blinded to virological and clinical metadata, including whether participants were influenza PCR+ or PCR-, during blood sample processing and determination of BCG lux growth.<br>Transcriptional profiling, cytokine quantification and flow cytometry: Investigators were blinded to clinical metadata, BCG lux growth and (where applicable) influenza PCR status, during sample processing and data acquisition.

Reporting for specific materials, systems and methods

We require information from authors about some types of materials, experimental systems and methods used in many studies. Here, indicate whether each material, system or method listed is relevant to your study. If you are not sure if a list item applies to your research, read the appropriate section before selecting a response.

Materials & experimental systems

n/a	Involved in the study	
☐	☒	Antibodies
☒	☐	Eukaryotic cell lines
☒	☐	Palaeontology and archaeology
☒	☐	Animals and other organisms
☒	☐	Clinical data
☒	☐	Dual use research of concern
☒	☐	Plants

Methods

n/a	Involved in the study	
☒	☐	ChIP-seq
☐	☒	Flow cytometry
☒	☐	MRI-based neuroimaging

Antibodies

Antibodies used	Antibodies used were in the form of commercial MSD assays and commercial fluorescence-conjugated antibodies for flow cytometry (details in methods)

1) MSD:
U-PLEX Custom Biomarker Group 1 (hu) Assays (Kit Catalog K15067L-1)with the following antibodies.
Human IFN-γ antibody set (Cat. B21TT-2)
Human TNF-α antibody set (Cat. B21UC-2)
Human IL-1β antibody set (Cat. B21TU-2)
Human IL-10 antibody set (Cat. B21TZ-2)
Human IL-17A/F antibody set (Cat. B21VY-2)
Human IL-22 antibody set (Cat. B21WI-2)
Human IL-23 antibody set (Cat. B21WG-2)

Analytes were measured across two plates, with one plate containing TNF-α, IFN-γ and IL-1β and the other plate containing IL-10, IL-17A/F, IL-22, IL23. All samples were added neat, except for the 6 h, 24 h and 72 h samples on the TNF-α, IFN-γ and IL-1β plate, which were diluted 1:10 with the included kit assay diluent. The manufacturers' instruction were followed in full.

The following S-PLEX kits were used:
S-PLEX Human IFN-α2a kit (Cat. K151P3S, Lot numbers K00S0070)
S-PLEX Human IFN-β kit (Cat. K151ADRS, Lot numbers K00S0081)
All samples were added neat. The manufacturers' instruction were followed in full.

2) Flow cytometry:
Biolegend FITC anti-human CD3 antibody, UCHT1 clone (Cat. 300405).
Biolegend PerCP-Cy5.5 anti-human CD4 antibody, OKT4 clone (Cat. 317427).
Biolegend PE anti human IL-10 antibody, JES3-9D7 clone (Cat. 501403).
Biolegend Alexa Fluor 647 anti human IFN-γ antibody, 4SB3 clone (Cat. 502516).
Biolegend Alexa Fluor 700 anti human CD14 antibody, 63D3 clone (Cat. 367113).
Biolegend Brilliant violet 605 anti human CD16 antibody, 3G8 clone. (Cat. 302039).
Biolegend Brilliant violet 711 anti human CD8a antibody, RPA-T8 clone (Cat. 301043)
Biolegend Brilliant violet 421 anti human TNFα antibody, Mab11 clone (Cat. 502931)
Biolegend Brilliant Violet 785 anti human HLA-DR antibody, L243 clone (Cat. 307641).
BD biosciences BV510 anti human CD56 antibody, NCAM16.2 clone (Cat. 563041).
Miltenyi Biotec PE-Vio 615 anti human IFN-α REAfinity antibody, REA1013 clone (Cat. 130-116-995).
All antibody dilutions were 1:100 except for PE anti -human IL-10 antibody which was 1:200.

Validation

1) Validation details are available for these antibodies on MSD's website, https://www.mesoscale.com/en/technical_resources/search_all_documents?document-type=datasheets. The website also states: MSD's validated assay kits meet the Clinical Laboratory Standards Institute guidelines for consistency, sensitivity, precision, and robustness. Validation testing is conducted through a design-control process according to the principles outlined in "Fit-for-Purpose Method Development and Validation for Successful Biomarker Measurement" by Lee, J.W. et al, available here: https://www.mesoscale.com/en/products_and_services/assay_kits/validated_kits.
We performed optimisation experiments prior to their use in this study.

An internal positive control was included on every plate. Optimal sample concentration for each analyte and timepoint was evaluated by earlier optimisation experiments.

2) Biolegend states "All of our products undergo industry-leading rigorous quality control (QC) testing to ensure the highest level of performance and reproducible results. Each lot is compared to an internally established "gold standard" to maintain lot-to-lot consistency. We also conduct wide-scale stability studies to guarantee an accurate shelf-life for our products", available here:https://www.biolegend.com/en-us/quality/quality-control. Details of validations and citations are available on the website per antibody product.

BD Biosciences state: "The specificity is confirmed by using multiple applications that may include a combination of flow cytometry, immunofluorescence, immunohistochemistry or western blot to test a combination of primary cells, cell lines or transfectant models. All flow cytometry reagents are titrated on the relevant positive or negative cells. To save time and cell samples for researchers, pre-titrated test size reagents are bottled at an optimal concentration, with the best signal-to-noise ratio on relevant models. You can look up the Certificate of Analysis and the concentration of test-size human reagents from specific lots via the Concentration Lookup page or BD Regulatory Documents. Technical data sheets provide data generated on the relevant primary model at this optimal concentration based on a titration curve. QC data on any lot of reagent can be requested through ResearchApplications@bd.com", available here: https://www.bdbiosciences.com/en-gb/products/reagents/flow-cytometry-reagents/research-reagents/quality-and-reproducibility. Details of validations and citations are available on the website per antibody product.

Miltenyi Biotec provides details of its quality and validations processes here: https://www.miltenyibiotec.com/GB-en/products/macs-antibodies/antibody-reproducibility-and-validation.html, with details of validations and citations available on the website per antibody product.

Control samples had previously been used to evaluate appropriate staining controls of antibody and fluorochrome interactions and spectral overlap.

Plants

Seed stocks

Report on the source of all seed stocks or other plant material used. If applicable, state the seed stock centre and catalogue number. If plant specimens were collected from the field, describe the collection location, date and sampling procedures.

Novel plant genotypes

Describe the methods by which all novel plant genotypes were produced. This includes those generated by transgenic approaches, gene editing, chemical/radiation-based mutagenesis and hybridization. For transgenic lines, describe the transformation method, the number of independent lines analyzed and the generation upon which experiments were performed. For gene-edited lines, describe the editor used, the endogenous sequence targeted for editing, the targeting guide RNA sequence (if applicable) and how the editor was applied.

Authentication

Describe any authentication procedures for each seed stock used or novel genotype generated. Describe any experiments used to assess the effect of a mutation and, where applicable, how potential secondary effects (e.g. second site T-DNA insertions, mosiacism, off-target gene editing) were examined.

Flow Cytometry

Plots

Confirm that:

☒ The axis labels state the marker and fluorochrome used (e.g. CD4-FITC).

☒ The axis scales are clearly visible. Include numbers along axes only for bottom left plot of group (a 'group' is an analysis of identical markers).

☒ All plots are contour plots with outliers or pseudocolor plots.

☒ A numerical value for number of cells or percentage (with statistics) is provided.

Methodology

Sample preparation

Whole blood was diluted 50:50 with RPMI/glutamine and 390 µl aliquots incubated with and without BCG lux. On removal from the incubator, 9 µl of 0.1 M liquid EDTA and 8.5 ml alternative lysis solution (ammonium chloride lysing solution) were added, the sample vortexed and incubated at room temperature for 10 minutes. After centrifugation at 1400 rpm for 5 minutes, the supernatant was poured off, the cells washed in PBS and 100 µl of 1% Zombie NIR Fixable Viability kit (BioLegend) then added, followed by a 20-minute incubation in the dark at 4 C°. After washing the cells, they were fixed with paraformaldehyde and cryopreserved (using 1 ml 10% DMSO and 25% heat inactivated fetal calf serum (FCS) in RPMI with L-glutamine) before storage at -80 °C.
Cryopreserved cells were thawed, washed, and permeabilized with Perm/wash solution (BD Biosciences). Cells were then incubated at 4°C for 1hour with fluorescence-conjugated antibodies directed against surface antigens and intracellular cytokines. The following fluorescence-conjugated anti-human antibodies were used: anti-CD3 FITC, anti-CD4 PerCP-Cy5.5, anti-CD8a Brilliant Violet 711, anti-CD14 Alexa Fluor 700, anti-CD16 Brilliant Violet 605, anti-CD56, anti-TNF-α Brilliant Violet 421, anti-IL10 PE, anti-IFN-γ Alexa Fluor 647, and anti-HLA-DR Brilliant Violet 785 (all BioLegend); anti-CD56 BV510 (BD Biosciences); anti-IFN-α PE-Vio 615 REAfinity (Miltenyi Biotec). Cells were washed and resuspended in FACS buffer (PBS, 2% FCS, 2µM EDTA) for acquisition.

Instrument

The entire sample was acquired on a BD LSR Fortessa Flow Cell Analyzer (BD Biosciences) at Imperial College London.

Software

For sample acquisition, FACSDiva software (version 9.2, BD Biosciences) was utilised. Compensation was performed, for overlap of fluorescence detection, using compensation beads (Biolegend Cat. 424602) . Control samples had previously been used to evaluate appropriate staining controls of antibody and fluorochrome interactions and spectral overlap. Data were analysed using FlowJo (version 10.10.0).

Cell population abundance

No sorting was performed for this study.

Gating strategy

Cells were gated using FSC-A/SSC-A, with dead cells and doublets excluded based on viability staining and FSC-H/FSC-A. Total lymphocytes were identified as CD3+ and further classified into CD4+ helper and CD8+ cytotoxic T cells. NK cells were defined by CD3-CD56+ expression. Total monocytes were identified as CD3-CD14+ cells, with subsets distinguished by CD14 and CD16 expression: classical (CD14++CD16-), intermediate (CD14++CD16++), and non-classical (CD14+CD16++). Cytokine expression (IFN-α, IFN-γ, TNF-α and IL-10) and HLA-DR expression were assessed across all cell subsets. The gating strategy is provided in Figure 6A.

☒ Tick this box to confirm that a figure exemplifying the gating strategy is provided in the Supplementary Information.
