# Supplementary material for: Influenza coinfection inhibits control of mycobacterial infection in a human challenge model
Source: Nat Commun. 2026 Jun 11;17:4884. doi: 10.1038/s41467-026-72363-2 (PMC13261052; doi:10.1038/s41467-026-72363-2)
Supplement: Supplementary file 24 — Transparent Peer Review file [file 41467_2026_72363_MOESM24_ESM.pdf]

nature portfolio

Peer Review File

Influenza coinfection inhibits control of mycobacterial infection in a human challenge model

Corresponding Author: Dr Claire Broderick

This file contains all reviewer reports in order by version, followed by all author rebuttals in order by version.

Version 0:

Reviewer comments:

Reviewer #1

(Remarks to the Author)

In this study the authors exploited an influenza human challenge model to further interrogate the link between influenza and TB. Both are major killers in the global south and so the work is of high significance.

1. The broader implications of this work, (e.g. seasonal influenza vaccination) and perhaps other interventions (including non-pharmaceutical ones) could be further emphasized for impact. The findings underscore that basic aspects such as better ventilation, use of masks in people who are coughing, better ventilation in taxis and public transport and schools, should be prioritized (to reduce influenza transmission) which will also benefit TB. More specifically, the study findings together with the epidemiological findings support the need for an interventional trial of a vaccination strategy the investigators propose. These points should be emphasized and expanded for impact.
2. A major limitation that should be mentioned is that the authors used BCG and not a virulent MTB strain like H37RA or a clinical strain. The findings could have been different.
3. A further limitation that should be mentioned in the discussion is the small sample sizes in the restrictors versus non-restrictors. So, type 2 error.
4. The figures are well outlined and the text flows well.
5. While the compartment-specific limitation has been mentioned, this could be spelled out more clearly. Sticking with that thought, in the 4th last paragraph, last sentence, it's a good suggestion to propose exploiting human BCG lung challenge models. The relevant missing reference here after having a quick look (something I came across recently) is the following reference which should be cited. This might be an excellent coinfection model to study what the authors propose. After having a quick look add the following ref:
https://pubmed.ncbi.nlm.nih.gov/31860339/
6. In line 344 the authors are suggesting that type 1 interferon responses may be linked to suboptimal mycobacterial control in their study. The authors have done a good job of summarising the type 1 interferon literature on TB and like most things in immunology, there were discordant findings with regard to whether the type 1 interferon response is helpful or deleterious to mycobacterial control? However, context is critical in immunology. What the authors have not done is contextulise their findings relative to these discordant findings in the literature. I think this will be most helpful for the readers and the field. In other words, what aspect of the discordance does the influenza human challenge model here with MGIA support? This will be a nice way to end that specific paragraph. So, contextualising in terms of the author's findings and giving the reader some insight and interpretation would be most useful.
7. In the methods section it is clear that this was a nasal challenge study. However, it remains unclear whether in the broader parent study lung samples by bronchoalveolar lavage were also obtained? This needs to be clarified.

(Remarks on code availability)

Reviewer #2

(Remarks to the Author)

In the manuscript entitled “ Influenza coinfection inhibits control of mycobacterial infection in a 1 human challenge model” the authors describe a study in which they took whole blood from individuals before and after challenge with H3N2 Influenza and to do a mycobacterial growth inhibition assay (MGIA). 30 people were challenged with influenza, 24 of whom were infected and 6 who were not. They compared the assays of those infected with those who were not. They demonstrated that after challenge with H3N2, the ability of the cells from infected individuals to control the growth of Mtb was significantly impaired. This was not true of those individuals who were not infected with influenza. They also demonstrated a differential gene expression profile in the cells of the individuals who were infected with influenza in the MGIA. The genes that were particularly affected were along the interferon pathway. The data from this study demonstrate that influenza inhibits the host’s ability to produce interferon, leading to lack of control of mycobacterial growth. The manuscript is well written, and provides a unique opportunity to explore the interplay of 2 common infections. It also provides mechanistic evidence for a phenomenon that has been observed epidemiologically, that of increased tuberculosis after influenza infection.

I have no suggestions for changes.

(Remarks on code availability)
I am able to access the code without difficulty, but I am not qualified to review the code for this manuscript.

Open Access This Peer Review File is licensed under a Creative Commons Attribution 4.0 International License, which permits use, sharing, adaptation, distribution and reproduction in any medium or format, as long as you give appropriate credit to the original author(s) and the source, provide a link to the Creative Commons license, and indicate if changes were made.
In cases where reviewers are anonymous, credit should be given to 'Anonymous Referee' and the source.
The images or other third party material in this Peer Review File are included in the article’s Creative Commons license, unless indicated otherwise in a credit line to the material. If material is not included in the article’s Creative Commons license and your intended use is not permitted by statutory regulation or exceeds the permitted use, you will need to obtain permission directly from the copyright holder.
To view a copy of this license, visit https://creativecommons.org/licenses/by/4.0/

“Influenza coinfection inhibits control of mycobacterial infection in a hum an challenge model”

Response to Reviewers

Dear Reviewers,

Thank you for taking the time to review our manuscript and for providing us with your valuable feedback. We are very grateful for your insightful comments and have considered them all carefully. We have incorporated your suggestions into the manuscript and have highlighted them in track changes. We provide a point-by-point response to your comments below. All page numbers refer to the revised manuscript.

<u>Responses to Reviewer 1:</u>

In this study the authors exploited an influenza human challenge model to further interrogate the link between influenza and TB. Both are major killers in the global south and so the work is of high significance.

1. The broader implications of this work, (e.g. seasonal influenza vaccination) and perhaps other interventions (including non-pharmaceutical ones) could be further emphasized for impact. The findings underscore that basic aspects such as better ventilation, use of masks in people who are coughing, better ventilation in taxis and public transport and schools, should be prioritized (to reduce influenza transmission) which will also benefit TB. More specifically, the study findings together with the epidemiological findings support the need for an interventional trial of a vaccination strategy the investigators propose. These points should be emphasized and expanded for impact.

Thank you for recognising the significance of these findings and for making this suggestion, we agree this point should be emphasised. We have added a paragraph towards the end of the discussion (lines 354-357).

2. A major limitation that should be mentioned is that the authors used BCG and not a virulent MTB strain like H37RA or a clinical strain. The findings could have been different.

We agree, this is a very important limitation to include. We have added this to our discussion on the limitations (lines 321-323).

3. A further limitation that should be mentioned in the discussion is the small sample sizes in the restrictors versus non-restrictors. So, type 2 error.

We agree, this limitation should be included. We have added this to our discussion on the limitations (lines 316-320).

4. The figures are well outlined and the text flows well.

Thank you very much for these very positive comments.

5. While the compartment-specific limitation has been mentioned, this could be spelled out more clearly. Sticking with that thought, in the 4th last paragraph, last sentence, it's a good suggestion to propose exploiting human BCG lung challenge models. The relevant missing reference here after having a quick look (something I came across recently) is the following reference which should be cited. This might be an excellent coinfection model to study what the authors propose. After having a quick look add the following ref: https://pubmed.ncbi.nlm.nih.gov/31860339/

Thank you for these comments and for finding and providing this reference, we agree that lung challenge models would provide an excellent opportunity to study coinfection. We have expanded this section in the Discussion to make this clearer and we have added this reference (lines 332-336).

6. In line 344 the authors are suggesting that type 1 interferon responses may be linked to suboptimal mycobacterial control in their study. The authors have done a good job of summarising the type 1 interferon literature on TB and like most things in immunology, there were discordant findings with regard to whether the type 1 interferon response is helpful or deleterious to mycobacterial control? However, context is critical in immunology. What the authors have not done is contextulise their findings relative to these discordant findings in the literature. I think this will be most helpful for the readers and the field. In other words, what aspect of the discordance does the influenza human challenge model here with MGIA support? This will be a nice way to end that specific paragraph. So, contextualising in terms of the author's findings and giving the reader some insight and interpretation would be most useful.

Thank you for this excellent suggestion. We have extended this paragraph so as to include this contextualisation (lines 281-286).

7. In the methods section it is clear that this was a nasal challenge study. However, it remains unclear whether in the broader parent study lung samples by bronchoalveolar lavage were also obtained? This needs to be clarified.

We have added the sentence "As per its protocol, NCT02755948 collected bronchoalveolar lavage samples from participants, but these did not contribute to this study" (lines 381-382).

Responses to Reviewer 2:

In the manuscript entitled " Influenza coinfection inhibits control of mycobacterial infection in a 1 human challenge model" the authors describe a study in which they took whole blood from individuals before and after challenge with H3N2 Influenza and to do a mycobacterial growth inhibition assay (MGIA). 30 people were challenged with influenza, 24 of whom were infected and 6 who were not. They compared the assays of those infected with those who were not. They demonstrated that after challenge with H3N2, the ability of the cells from infected individuals to control the growth of Mtb was significantly impaired. This was not true of those individuals who were not infected with influenza. They also demonstrated a differential gene expression profile in the cells of the individuals who were infected with influenza in the MGIA. The genes that were

particularly affected were along the interferon pathway. The data from this study demonstrate that influenza inhibits the host's ability to produce interferon, leading to lack of control of mycobacterial growth. The manuscript is well written, and provides a unique opportunity to explore the interplay of 2 common infections. It also provides mechanistic evidence for a phenomenon that has been observed epidemiologically, that of increased tuberculosis after influenza infection.
I have no suggestions for changes.

Reviewer #2 (Remarks on code availability):
I am able to access the code without difficulty, but I am not qualified to review the code for this manuscript.

Many thanks for your very positive comments about our study and manuscript. We are pleased you were able to access the code easily.
